# Using comparative genomics to understand molecular features of carbapenem-resistant *Acinetobacter baumannii* from South Korea causing invasive infections and their clinical implications

**DOI:** 10.1371/journal.pone.0229416

**Published:** 2020-02-21

**Authors:** Min Hyung Kim, Haeyoung Jeong, Young Mi Sim, Soohyun Lee, Dongeun Yong, Choong-Min Ryu, Jun Yong Choi

**Affiliations:** 1 Department of Internal Medicine, Bundang Jesaeng Hospital, Seongam, Gyeonggi, South Korea; 2 Department of Internal Medicine and AIDS Research Institute, Yonsei University College of Medicine, Seoul, South Korea; 3 Infectious Disease Research Center, KRIBB, Daejeon, South Korea; 4 R&D Center, Medytox Inc., Suwon, Gyeonggi-do, South Korea; 5 Department of Laboratory Medicine and Research Institute of antimicrobial resistance, Yonsei University College of Medicine, Seoul, South Korea; Zhejiang University, CHINA

## Abstract

*Acinetobacter baumannii* is a highly potent nosocomial pathogen that is associated with increased in-hospital mortality. Here, we investigated the changes in molecular characteristics of carbapenem-resistant *A*. *baumannii* (CRAB) isolated from the blood samples of patients admitted to a tertiary hospital in South Korea from January 2009 to July 2015. Whole genome sequencing using the Illumina MiSeq platform and multi-locus sequence typing (MLST) were performed for 98 CRAB clinical isolates. In silico analyses for the prediction of antimicrobial resistance and virulence factor genes were performed. Plasmid sequences, including complete forms, were reconstructed from the sequence reads. Epidemiologic data were collected from the hospital database. MLST using the Oxford scheme revealed 10 sequence types of CRAB, of which ST191 was the dominant type (n = 59). Although *bla*_OXA-23_ was shared by most analysed strains, the compositions of antimicrobial resistance determinants differed among sequence types. ST447 and ST451/ST1809 with a few resistance genes were isolated during the later years of the study period. The number of virulence genes increased, while that of ST191 did not change significantly over the investigation period. Intriguingly MLST types, compositions of antimicrobial resistance genes, and virulence genes had no association with clinical outcomes of CRAB bacteraemia. In conclusion, active changes in or accumulations of antimicrobial resistance determinants and virulence genes in CRAB were not observed during the research period. Molecular characteristics of CRAB had no association with clinical outcomes of CRAB bacteraemia.

## Introduction

*Acinetobacter baumannii* is an aerobic gram-negative coccobacillus known for relatively few virulence factors as compared to other gram-negative pathogens [1]. Its ability to acquire various antimicrobial resistance genes makes it a highly successful nosocomial pathogen, which is associated with increased in-hospital mortality [2]. In particular, the prevalence of carbapenem-resistant *A*. *baumannii* (CRAB) isolates is on the rise in Asian countries, including South Korea [3,4]. The specific clone, international clone 2 (IC II), is a major clonal group among Korean CRAB isolates [5]. This organism is associated with nosocomial outbreaks and multidrug resistance in association with *bla*_OXA-23-like_ genes [6]. In a recent study, 75% of *A*. *baumannii* clinical isolates were shown to belong to IC II in Korea [7], and IC II was recognised as the second most common cause of bacteraemia from 2012 to 2013 [8].

Considering the threat of the increasing incidence of CRAB infection, prediction of future effects of these isolates is a priority. Whole genome sequencing (WGS) analysis provides unprecedented information about this illusive organism, especially for the characterisation of nosocomial outbreaks [9,10]. WGS provides in-depth epidemiologic knowledge by providing higher resolution in genotyping and suggesting the emergence of new epidemic clones. WGS also helps researchers to understand the various mechanisms underlying the acquisition of antimicrobial resistance genes. Antimicrobial resistance genes were found in alien islands of accessory genomes and were often accompanied by integrases, transposases, or insertion sequences suggestive of their possible acquisition by horizontal gene transfer from other *Acinetobacter* spp. or bacteria that colonise the same environment [11,12]. Furthermore, although little is known about the virulence factors of this bacterium as compared to the current knowledge about antimicrobial resistance mechanisms [13], the rapid increase in the number of sequenced and annotated genomes has enabled comparative genomic analyses to elucidate clues concerning potential virulence factors.

Previous studies compared the distribution of genes related to virulence and antimicrobial resistance to understand phenotypic differences between isolates [14–16]. However, a comprehensive analysis of the number of virulence or AMR genes in association clinical outcomes has not been performed. This analysis may provide some insight into how this organism is evolving in epidemiologic perspective. Therefore, we attempted a whole genome-association study by sequencing all available isolates from patients with CRAB bacteraemia to characterise the genomic changes over the study period from the epidemiological perspective and investigated the clinical significance of genomic characteristics of CRAB. These investigations of the genetic characteristics, including antimicrobial resistance determinants and virulence factors of CRAB blood isolates, can over time provide insight into how the organism evolves and may suggest clinico-epidemiological implications of genomic characteristics of this organism.

## Materials and methods

### Ethics statement

This study was approved by the Institutional Review Board (IRB) of Yonsei University Health System Clinical Trial Center (4-2017-0730). Since the study had minimal health risk and the study subjects were anonymised, the Institutional Review Board waived the requirement for written informed consent from the patients.

### Collection of bacterial isolates and patient information

Blood isolates from patients with CRAB bacteraemia were collected between January 2009 and July 2015 at intensive care units (ICUs) of Severance Hospital in Seoul, Republic of Korea. CRAB bacteraemia was defined as one or more positive blood cultures for carbapenem-resistant *A*. *baumannii* and the presence of clinical features compatible with systemic inflammatory response syndrome. Preliminary species identification and antimicrobial susceptibility tests were conducted with VITEK II system (bioMérieux, Marcy l'Etoile, France). Disc-diffusion testing using antimicrobial discs (Becton Dickinson, Sparks, MD, USA) on cationic-adjusted Mueller–Hinton (MH) agar (Difco Laboratories, Detroit, MI, USA) was subsequently performed as per CLSI guidelines [17]. Minimum inhibitory concentrations (MICs) to three drugs including tigecycline, minocycline and colistin were assessed using VITEK II system. The MICs of imipenem and meropenem were determined with the agar dilution method using MH agar following CLSI guidelines [17]. Additionally, modified Hodge tests and double-disk synergy tests were performed to screen carbapenemase and metallo-β-lactamase activity, respectively.

We collected clinical data, including patients’ age and sex, length of ICU stay, length of hospital stay, pre-existing chronic comorbidities (diabetes, chronic heart failure, chronic liver disease, chronic renal disease, and chronic pulmonary disease), sequential organ failure assessment score, episodes of hospital admission, previous invasive procedures (central line insertion, intubation, continuous renal replacement therapy, and surgery under general anaesthesia), as well as lengths and types of antibiotic treatments. The origin of bacteraemia was determined upon identification of precedent CRAB isolation or evidence of infection before the event of bacteraemia.

### Whole genome sequencing (WGS), annotation, prediction of antimicrobial resistance determinants, and identification of virulence genes

The isolates were cultured in Luria Bertani (LB) broth, and genomic DNA was extracted with a PureHelix™ Genomic DNA Prep Kit (cat. no. GCTN100, Nanohelix, Daejeon, Republic of Korea). Paired end sequencing of 98 *A*. *baumannii* clinical isolates was carried out using the Illumina MiSeq platform at ChunLab (Seoul, Republic of Korea). Specifically, TruSeq Nano DNA Library Preparation Kit (550 bp library size) and MiSeq Reagent Kit v3 (600 cycles) were used. Using CLC Genomics Workbench v11.0.1 (https://www.qiagenbioinformatics.com/), reads were trimmed (quality limit 0.01, ambiguous limit 1, min length 120 bp, discarding orphan reads) and were *de novo* assembled. Word and bubble sizes were 64 and 100, respectively. The assembled genome sequences were annotated using NCBI’s Prokaryotic Genome Annotation Pipeline (PGAP) v4.5 [18]. GenBank files produced from PGAP were converted to GFF format, and then subjected to Roary [19] to obtain core genome sequences and their codon-aware alignments, followed by a heuristic method-based automated trimming using trimAl [20]. A phylogenetic tree was constructed using FastTree2 [21] and visualised using iTOL server [22].

To identify antimicrobial resistance genes, the contig sequences were queried against the ResFinder database (https://cge.cbs.dtu.dk/services/data.php; downloaded on June 4, 2018) using Find Resistance v1.01 from the Microbial Genomics Module of CLC Genomics Workbench.

A comprehensive list of virulence factors from *A*. *baumannii* was compiled as per the previous studies [23–26]. The list of 182 virulence genes is shown in S1 Table. Distribution of virulence genes across all genomes was obtained using the large-scale blast score ratio (LS-BSR) [27]. A cut-off value of blast score ratio 0.7 was arbitrarily selected to evaluate the existence of each virulence gene.

For comprehensive genomic epidemiological analysis, we used BacWGSTdb (http://bacdb.org/BacWGSTdb) [28] which allows us to find closest isolates that are currently deposited in NCBI GenBank database. Scheme core genome multi-locus sequence typing (cgMLST) was used with a 50 threshold for single nucleotide polymorphism (SNP) and a 50 threshold for MSLT.

### Multi-locus sequence typing (MLST) and detection of single nucleotide polymorphism (SNP)

Allelic profiles or STs of each isolate were determined by submitting contig sequences to the PubMLST site (https://pubmlst.org/abaumannii/) according to the Institute Pasteur scheme (MLST-IP) and Oxford Database (MLST-OD).

SNPs were identified by mapping raw sequencing reads to the complete chromosome sequence of *A*. *baumannii* strain KBN10P02143 (GenBank CP013924.1), the multidrug strain that completely sequenced for the first time in South Korea, using Snippy (https://github.com/tseemann/snippy).

### Analysis of plasmid sequences

Plasmid sequences were constructed either by plasmidSPAdes [29] assembler that uses the read coverage of contigs, or by a custom three-step method consisting of (i) running Plasmid Profiler [30] that first identifies putative plasmid hits from the plasmid database, (ii) mapping of the entire reads to chosen plasmid sequences using SMALT (https://www.sanger.ac.uk/science/tools/smalt-0) and extracting paired reads where at least one end mapped using SAMTOOLS (http://www.htslib.org/), and finally, (iii) assembly of the extracted reads using UniCycler [31]. Assembly statistics and whole-genome assembly results are shown in S2 Table. Complete (also circularised) plasmid sequences were identified from UniCycler assemblies by visualization of assembly graphs using Bandage [32]). Putative protein coding genes were predicted using GeneMarkS [33], and *rep* genes were identified by hmmsearch (http://hmmer.org/) against Pfam database v31.0 (https://pfam.xfam.org/).

### Identification of insertion sequences and transposons

The *bla*_OXA-23_ is a major determinant of nosocomial outbreaks of IC II CRAB. The expression of this gene is presumably regulated by several insertion sequences, including IS*Aba1*, which is thought to play the most crucial role in gene expression [34]. ISMapper [35] was used to identify IS*Aba1* insertion sites on the reference genome of the strain KBN10P02143. Insertion sites of IS*Aba1* relative to the chromosome of KBN10P02143 are shown in S3 Table. The presence of *bla*_OXA-23_-carrying transposons, including Tn*2006*, Tn*2007*, Tn*2008*, and Tn*2009*, was screened using Primersearch from the EMBOSS package (http://emboss.sourceforge.net/), with primer sets suggested by Chen *et al*. [36].

### Statistical analysis

Baseline characteristics were compared using Mann–Whitney U test or independent samples *t*-test for continuous variables, and by χ^2^ test or Fisher’s exact test for categorical variables. Continuous variables were expressed as means or medians (interquartile ranges), while categorical variables were expressed as numbers with percentages for the description of baseline characteristics. Statistical analyses were performed using SPSS version 21.0 (IBM Corp., Armonk, NY, USA).

### Data availability

Genome sequences were submitted to GenBank under BioProject no. PRJNA448358 (https://www.ncbi.nlm.nih.gov/bioproject/PRJNA448358). The complete list of strain names and accession numbers are shown in S2 Table. Complete sequences of five representative plasmids (pABAY04001_1A, pABAY09008_1B, pABAY10001_1C, pABAY14012_4D, and pABAY15001_6E) are available from GenBank under the accession numbers MK386680.1- MK386684.1. All 115 complete plasmid sequences and their *rep* genes are available at https://doi.org/10.6084/m9.figshare.11467023.v1 (figshare).

## Results

### Overview of *A*. *baumannii* isolates

A total of 109 CRAB blood isolates were used in this study. Of these, three samples with possible contamination and eight samples with insufficient clinical data were excluded. The remaining 98 samples were consequently included in the analysis. The isolates exhibited a high rate of resistance for almost all the antibiotics tested: 92.7% were resistant to gentamycin (89/96), 88.8% to amikacin (87/98), 100% to imipenem (98/98), 98.8% to ciprofloxacin (84/85), 97.8% to levofloxacin (96/98), 98.9% to piperacillin-tazobactam (92/93), 98.9% to ceftazidime (96/97), 96.9% to cefepime (95/98), 4.3% to colistin (4/92), and 11.1% to tetracycline (1/9) (S4 Table).

### MLST analysis depending on isolation years

An MLST using Pasteur scheme identified 93 strains (94.9%) that were classified into ST2, indicating that most of the strains belonged to IC II. MLST using Oxford scheme was applied to assign 10 STs (ST858, ST369, ST208/ST1806, ST784, ST451/ST1809, ST357, ST368, ST447, ST552, and ST191). ST191 was the most abundant type (59 strains). Strains ABAY12003 and ABAY13017, single variants of ST191 at *gyrB* and *rpoD*, respectively, were not assigned any STs. As a result, we found that 91 strains (92.9%), all belonging to IC II, had two incidences of *gdhB* alleles, 3 and 189, which made distinctions between ST208, ST1806, ST451, and ST1809 ambiguous. Although all ST191 strains had two *gdhB* alleles, only the ABAY15007 strain harbouring *gpi*-94 *gpi*-107 was equivocally assigned to ST191 and ST784.

**Fig 1:**
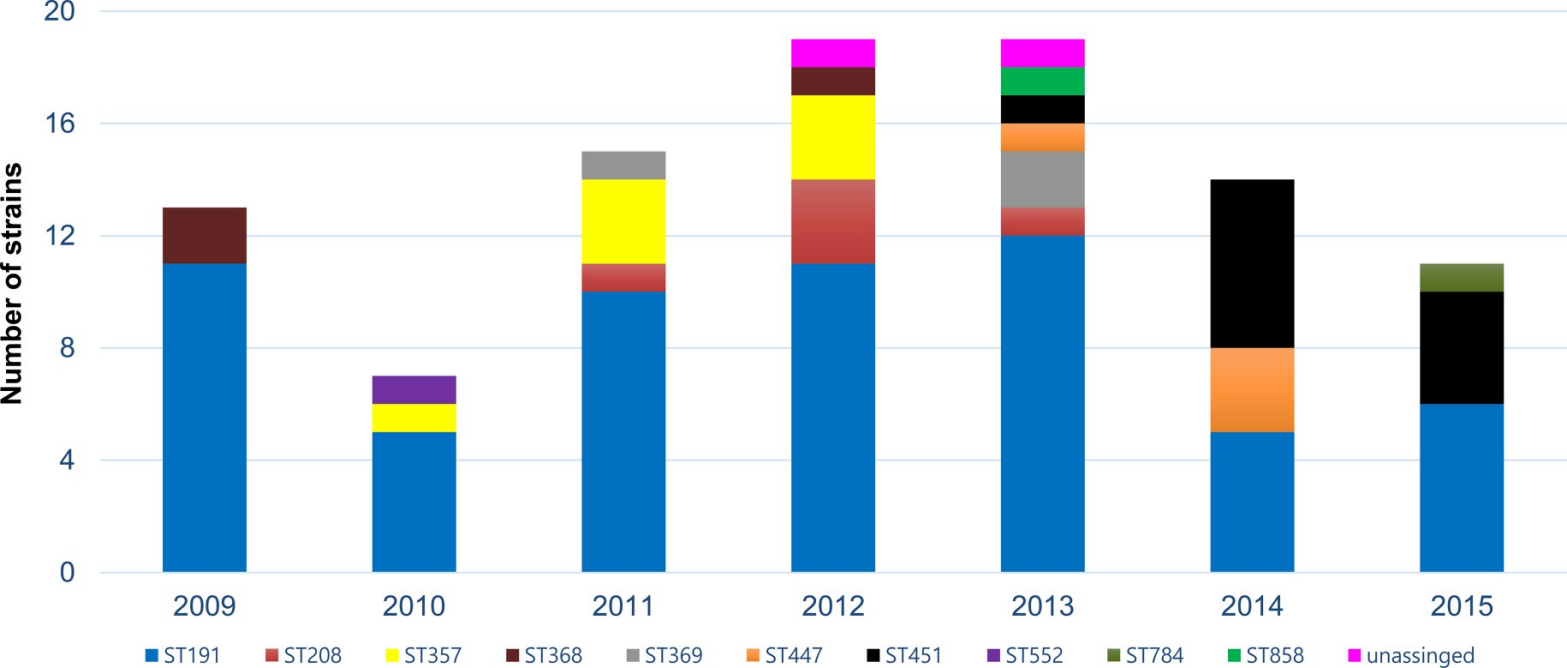
Distribution of carbapenem resistance *A*. *baumannii* isolates according to sequence types and year of isolation. The isolates were typed by multi-locus sequence typing (MLST) using Oxford scheme and colored individually according to sequence type. Bar graph represents the individual and cumulative numbers of strains isolated each year.

ST191 was evenly isolated throughout the research period. ST368 (n = 3), ST552 (n = 1), ST369 (n = 3), ST357 (n = 7), and ST208/ST1806 (n = 5) were mostly isolated from 2009 to 2012, while ST447 (n = 4), ST451/ST1809 (n = 11), ST858 (n = 1), ST784 (n = 1), and ST191/ST784 (n = 1) were mostly reported from 2013 to 2015 (Fig 1 and Table 1).

**Table 1:** Sequence types of carbapenem resistant *A*. *baumannii* (CRAB) and epidemiologic information.

Group (N)	Phylogenetic clade	Pasteur ST (N)	Oxford ST (N)	Isolated location (N)	Isolated year (N)	Origin of bacteremia
IC II	Clade 1	ST2 (93)	ST191 (59)	MICU A (15)	2009 (11)	Respiratory tract (46)
				MICU B (24)	2010 (5)	Gastrointestinal tract (3)
				MICU C (7)	2011 (10)	Musculoskeletal (1)
				MICU D (5)	2012 (11)	Catheter (6)
				NCU B (1)	2013 (12)	Other (2)
				SICU (2)	2014 (5)	
				CCU A (1)	2015 (5)	
				CCU B (2)		
	Clade 2		ST368 (3)	MICU B (2)	2009 (2)	Respiratory tract (3)
				CCU A (1)	2012 (1)	
	Clade 2		ST357 (7)	MICUA(5)	2010 (1)	Respiratory tract (7)
				MICUB(1)	2011 (3)	
				MICU D (1)	2012 (3)	
	Clade 3		ST208/ST1806 (5)	MICU B (2)	2011 (1)	Other (1)
				MICU C (1)	2012 (3)	
				MICU D (1)	2013 (1)	
	Clade 3		ST369 (3)	MICU A (1)	2011 (1)	Respiratory tract (2)
				MICU D (2)	2013 (2)	Other (1)
	Clade 2		ST858 (1)	MICU C (1)	2013 (1)	Respiratory tract (1)
	Clade 4		ST451/ST1809 (11)	MICU A (6)	2013 (1)	Respiratory tract (9)
				MICU B (4)	2014 (6)	Urinary tract(1)
				CCU A (1)	2015 (4)	Gastrointestinal tract (1)
	Clade 1		ST784 (1)	MICU C (1)	2015 (1)	Musculoskeletal (1)
	Clade 1		ST191/ST784 (1)	MICU B (1)	2015 (1)	Respiratory tract (1)
Non-IC II	NA	ST193 (1)	ST552 (1)	CCU A (1)	2010 (1)	Respiratory tract (1)
	NA	ST10 (4)	ST447 (4)	MICU A (2)	2013 (1)	Respiratory tract (4)
				MICU B (2)	2014 (3)	
NA	NA	NA	ABAY12003	MICU A (1)	2012 (1)	Respiratory tract (1)
			ABAY13017	MICU A (1)	2013 (1)	Catheter (1)

Data are expressed as number (N). Abbreviation: ST, sequence type; IC II, international clone II; MLST, Multi-locus sequence typing; MICU, medical intensive care unit; NCU, neurologic intensive care unit; SICU, surgical intensive care unit; CCU, cardiac intensive care unit; NA, not applicable.

### Core genome-based phylogeny and SNP-based inference of recombination

**Fig 2:**
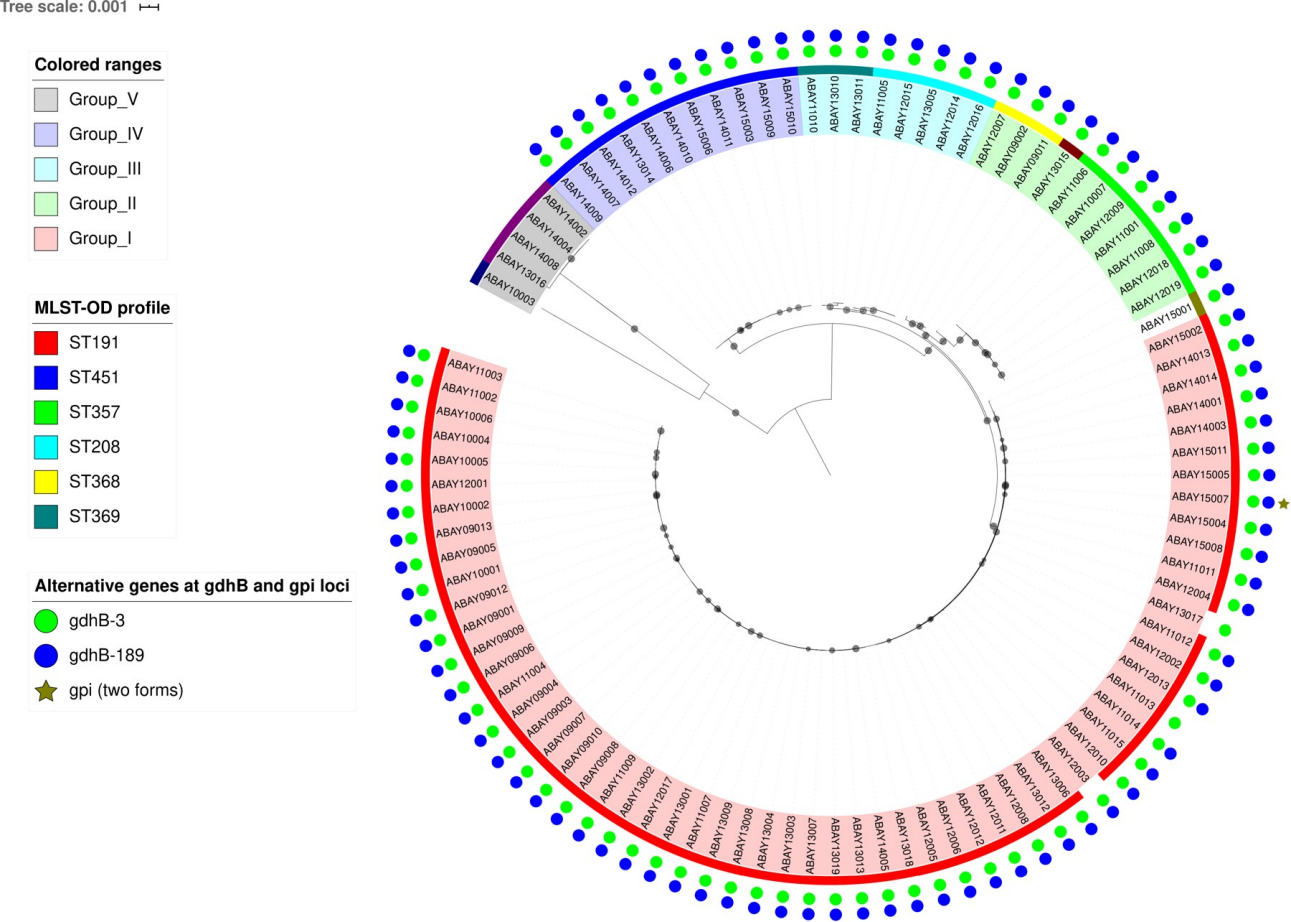
Core-gene based phylogenetic tree of 98 *A*. *baumannii* strains. Approximately maximum-likelihood tree was constructed from 2,520 core gene sequences (2,347,373 bp) using FastTree 2. Gray circles on branches represent local support values (0.9–1.0) based on Shimodaira-Hasegawa test.

A core genome alignment-based phylogenetic tree was constructed to delineate the in-depth relationships between analysed strains (Fig 2). The analysis resulted in the classification of 98 analysed strains into four major clades. SNP identification relied on read mapping on the reference genome sequence of strain KBN10P02143. Pairwise SNP distances among all strains ranged from 39 to 83,304 bp (5,368 bp median) (S5 Table). Relationships between SNP-based phylogeny and STs were searched. Clade 1 was mostly composed of ST191. The majority of strains classified as clade 2 (ST368, ST357, and ST858) were isolated in the former part of our study period, while those of clade 3 (ST208/ST1806 and ST369) were isolated in the latter part. However, chronological correlation among clades was not identified overall.

### Differences in patient characteristics among STs

Patients were hospitalised in different types of ICUs, and the locations of each isolated ST are shown in Table 1. Most strains were identified in MICU, and no notable relationship between isolated locations and STs was reported. The mean age of the enrolled patients was 60.62 ± 14.63 years, and male patients predominated (70.4%, n = 69). Chronic obstructive pulmonary disease was the most commonly found underlying morbidity (93.9%, n = 92), and chemotherapy was the most common cause of immunosuppression (39.8%, n = 39). Ventilator care was performed in 81.6% of patients (n = 80). The most common source of bacteraemia was the respiratory tract (79.6%, n = 78). The median number of hospitalisation days was 30 [19–64.2], and patients used antibiotics for a median treatment length of 30.5 [16–59.3] days. The all-cause 28-day mortality was 80.6% (n = 79). No significant differences were observed in clinical characteristics among STs except for the following. Patients with strains typed as ST447 Patients with strains typed as ST447 spent less time in the hospital (17 [17–41] days versus 30 [19–64.2] days, p < 0.05) and there were fewer males among patients with strains typed as ST191 (40 [63.5%] versus 29 [82.9%], p < 0.05) (Table 2).

**Table 2:** Baseline characteristics of patients with carbapenem resistant *A*.*baumannii* bacteremia, grouped by sequence types.

Chracteristics	Grouped by sequence type						
	ST191			ST447			Total
	ST191(N = 59)	Non ST191(N = 39)	p-value	ST447(n = 4)	Non ST447(n = 94)	p-value	
**Age, years**	59.27±14.70	63.13±14.39	0.21	77.0±10.74	60.09±14.41	0.05	60.62±14.63
**Gender, male**	**37(62.7)**	**32(84.2)**	**0.02**	3(75.0)	66(71.0)	0.67	69(70.4)
**Comorbidities**							
DM	43(72.9)	32(82.1)	0.23	2(50.0)	73(77.7)	0.24	75(67.5)
COPD	55(93.2)	37(94.9)	0.56	4(100.0)	88(93.6)	0.77	92(93.9)
CHF	49(83.1)	34(87.2)	0.42	4(100.0)	79(84.0)	0.51	83(84.7)
HTN	36(61.0)	26(66.7)	0.40	2(50.0)	60(63.8)	0.47	62(63.3)
CRF	42(71.2)	32(82.1)	0.18	3(75.0)	71(75.5)	0.69	74(75.5)
Chronic liver disease	52(88.1)	37(94.8)	0.24	4(100.0)	85(90.4)	0.60	89(90.8)
Malignancy	35(59.3)	22(56.4)	0.56	2(50.0)	55(58.5)	0.67	57(58.2)
**Immunosuppressed status**							
Transplantation	7(11.9)	8(21/1)	0.18	0(0.0)	15(16.1)	0.50	15(15.3)
Steroid use^a^	20(33.9)	10(26.3)	0.29	0(0.0)	30(32.3)	0.22	30(30.6)
Chemotherapy	23(39.0)	16(42.1)	0.46	2(50.0)	37(39.8)	0.53	39(39.8)
**APACHE II**	21.32±8.16	21.26±7.18	0.97	23.25±3.50	21.22±7.881	0.35	21.17±7.81
**Admission episode(≥ once)**	42(71.19)	25(64.10)	0.27	3(75.0)	64(68.1)	0.62	67(68.4)
**Source of infection**							
Respiratory tract	46(82.1)	32(88.9)	0.29	4(100.0)	74(78.7)	0.35	78(79.6)
Other	13(22.0)	7(17.9)		0(0.0)	20(21.3)		
**Time to bacteremia**	21(12–41)	19(12–41)	0.38	12(8.2–37)	20.5(12–41)	0.21	20.5(12–41)
**Invasive procedure**							
Ventilator	47(79.7)	33(86.8)	0.27	4(100.0)	76(81.7)	0.47	80(81.6)
CRRTx	18(30.5)	11(28.9)	0.53	2(50.0)	27(29.0)	0.35	29(29.6)
C-line insertion	52(88.1)	35(92.1)	0.40	4(100.0)	83(89.2)	0.64	87(88.8)
Operation	21(35.6)	8(21.1)	0.09	0(0.0)	29(31.2)	0.24	29(29.6)
**Hospital stay (days)**	32(20–57)	27(17–71)	0.77	**17(17–41)**	**30(19–64.2)**	**0.04**	30(19–64.2)
**ICU stay (days)**	22(11–38)	19(8–52)	0.34	17(15.5–33.5)	21(9.8–40.5)	0.34	21(9.8–40.5)
**Duration of antibiotics**	31(17–57)	25(16–69)	0.84	16.5(16–41)	30.5(16–59.3)	0.07	30.5(16–59.3)
**Duration of susceptible antibiotics**	0(0–9)	8(0–17)	0.36	4.5(0.3–10.8)	2(0–11.25)	0.76	2(0–11)
**Susceptible antibiotics use**^**b**^	4(6.8)	7(18.4)	0.08	1(25)	10(10.8)	0.39	11(11.2)
**All cause 28 day mortality**	52(88.1)	27(71.1)	0.16	4(100.0)	75(80.6)	0.81	79(80.6)

Data of sequence types only with statistical significance are shown. Data are expressed as the mean ± SD / median (Q1-Q3) or N (%). Abbreviation: DM, diabetes mellitus; COPD, chronic obstructive pulmonary disease; CHF, congestive heart failure; HTN, hypertension; CRF, chronic renal failure; APACHE, acute physiology and chronic health evaluation; CRRTx., continuous renal replacement therapy.

*signifies having statistical significance with p-value<0.05 when compared to groups of the rest.

^a^ Steroid use defined by use of ≥20mg prednisolone for ≥2weeks.

^b^ We consider antibiotics susceptible when phenotypically susceptible antibiotics were used for more than half of time after culture recovered.

Analysis using BacWGSTdb based on MLST strategy revealed that 48 (48/98, 49.0%) strains had no closely related strains. The other strains had close relationships with clinical isolates identified in South Korea. The only strain that was linked to isolates outside of South Korea was ABAY09002 (closely related to GeneBank JRQX01, isolated in Thailand in January, 2010). The rates of strains that showed no relatedness with previously identified strains were as follows: ST191, 40.0% (23/59); ST368, 66.6% (2/3); ST552, 100%(1/1); ST357, 71/43%(5/7); ST208/ST1806, 100%(5/5); ST369, 100%(3/3); ST451/ST1809, 9.1%(1/11); ST8958, 100%(1/1); ST784, 100%(1/1); ST191/ST784, 100%(1/1); ST447,25.0%(1/4); Not applicable (NA), 100%(2/2).

### Differences in antimicrobial resistance determinants according to STs and year of isolation

Among 29 AMR genes investigated, an average of 16.64 genes were possessed by each strain. The gene *bla*_OXA-23_ was identified in all strains except for ABAY11010 (ST369) and ABAY12016 (ST208/ST1806). Among class D carbapenem-hydrolysing class D β-lactamase genes, *bla*_OXA-82_ and *bla*_OXA-66_ were found in ABAY11010 and ABAY12016, respectively.

The composition of antimicrobial resistance determinants within each ST was relatively constant, especially in ST191. As we compared the first half of the research period with the latter half, only *strA* (0%, n = 0 versus 18.2%, n = 4; p = 0.02) showed increased prevalence. Furthermore, we observed a decrease in the prevalence of *aac*(3)-*Ia* (9.1%, n = 2 versus 91.9%, n = 34; p < 0.01), *armA* (86.4%, n = 19 versus 100%, n = 37; p = 0.04), and *bla*_TEM-1D_ (18.2%, n = 4 versus 97.3%, n = 36; p < 0.01). The comparison of sources of bacteraemia showed that *aadA1b* was more common in isolates from the respiratory tract than in isolates from any other sources (51.9%, n = 14 versus 0%, n = 0; p < 0.01) (Table 3).

**Table 3:** Composition of antimicrobial resistant genes of all sequence types combined and sequence type 191 according to isolation date or source of bacteremia.

Genes	ST191						All		
	Source of bacteremia			Isolated year			Isolated year		
	Respiratory tract(n = 46)	Other(n = 13)	p-value	2009-2012(n = 37)	2013-2015(n = 22)	p-value	2009-2012(n = 54)	2013-2015(n = 44)	p-value
**Aminoglycoside**									
*aac(3)-Ia*	29(63.0)	7(53.8)	0.39	**34(91.9)**	**2(9.1)**	**<0.01**	**38(70.4)**	**3(6.8)**	**<0.01**
*aac(6')-Iaf*	0(0.0)	0(0.0)	1.00	0(0.0)	0(0.0)	1.00	2(3.7)	0(0)	0.30
*aac(6')-Ib*	44(95.7)	13(100)	0.60	36(97.3)	21(95.5)	0.61	**50(92.6)**	**29(65.9)**	**<0.01**
*aac(6')-Ib3*	44(95.7)	13(100)	0.60	37(100)	20(90.9)	0.14	**51(94.4)**	**28(63.6)**	**<0.01**
*aadA1*	45(97.8)	13(100)	0.78	37(100)	21(95.5)	0.37	**51(94.4)**	**29(65.9)**	**<0.01**
*aadA1b*	**14(51.9)**	**0(0.0)**	**<0.01**	10(27.0)	4(18.1)	0.57	30(55.6)	17(68.6)	0.07
*ant(3'')-Ia*	44(95.7)	13(100)	0.60	37(100)	20(90.9)	0.14	**51(94.4)**	**28(63.6)**	**<0.01**
*aph(3')-Ia*	0(0.0)	0(0.0)	1.00	0(0.0)	0(0.0)	1.00	6(11.1)	9(20.5)	0.16
*aph(3'')-Ib*	0(0.0)	0(0.0)	1.00	0(0.0)	0(0.0)	1.00	14(25.9)	15(34.1)	0.26
*aph(6)-Id*	0(0.0)	0(0.0)	1.00	0(0.0)	0(0.0)	1.00	14(25.9)	15(34.1)	0.26
*aph(3')-VIb*	0(0.0)	0(0.0)	1.00	0(0.0)	0(0.0)	1.00	3(5.6)	1(2.3)	0.39
*aph(3')-Vij*	1(2.2)	0(0.0)	0.78	1(2.7)	0(0.0)	0.63	4(7.4)	1(2.3)	0.25
*armA*	43(93.5)	13(100)	0.47	**37(100)**	**19(86.4)**	**0.04**	51(94.4)	37(84.1)	0.09
*strA*	4(8.7)	0(0.0)	0.36	**0(0.0)**	**4(18.2)**	**0.02**	**14(25.9)**	**21(47.7)**	**0.02**
**b-lactam**									
*blaADC-25*	46(100)	13(100)	1.00	37(100)	22(100)	1.00	53(98.1)	40(90.9)	0.12
*blaOXA-23*	45(97.8)	13(100)	0.78	37(100)	21(95.5)	0.37	53(98.1)	43(97.7)	0.69
*blaOXA-66*	46(100)	13(100)	1.00	37(100)	22(100)	1.00	52(96.3)	39(90.7)	0.24
*blaOXA-68*	0(0.0)	0(0.0)	1.00	0(0.0)	0(0.0)	1.00	0(0)	3(6.8)	0.09
*blaOXA-82*	0(0.0)	0(0.0)	1.00	0(0.0)	0(0.0)	1.00	1(1.9)	2(4.5)	0.42
*blaOXA-120*	1(2.2)	0(0.0)	0.78	1(2.7)	0(0.0)	0.67	1(1.9)	0(0)	0.55
*blaPER-1*	0(0.0)	0(0.0)	1.00	0(0.0)	0(0.0)	1.00	3(5.6)	1(2.3)	0.39
*blaTEM-1D*	33(71.7)	7(53.8)	0.19	**36(97.3)**	**4(18.2)**	**<0.01**	**47(87.0)**	**12(27.3)**	**<0.01**
**Microlide**									
*mph(E)*	37(80.4)	13(100)	0.08	35(94.6)	15(68.2)	0.01	**49(90.7)**	**31(70.5)**	**0.01**
*msr(E)*	41(89.1)	12(92.3)	0.60	34(91.9)	19(86.4)	0.40	48(88.9)	37(84.1)	0.34
**Sulphonamide**									
*sul1*	45(97.8)	12(92.3)	0.40	37(100)	20(90.9)	0.14	**51(94.4)**	**28(63.6)**	**<0.01**
*sul2*	0(0.0)	0(0.0)	1.00	0(0.0)	0(0.0)	1.00	12(22.2)	17(38.6)	0.06
**Chloramphenicol**									
*catB8*	44(95.7)	12(92.3)	0.53	35(94.6)	21(95.5)	0.69	**49(90.7)**	**29(65.9)**	**<0.01**
**Quinolone**									
*aac(6')-Ib-cr*	40(86.9)	12(92.3)	0.35	45(97.8)	17(81.0)	0.12	**50(92.6)**	**29(65.9)**	**<0.01**
**Tetracycline**									
*tet(B)*	0(0.0)	0(0.0)	1.00	0(0.0)	0(0.0)	1.00	**11(20.4)**	**18(40.9)**	**0.02**

Data are expressed as N (%). Values with statistical significance (p-value<0.05) are expressed in boldface.

However, we observed clear distinctions in the composition of AMR genes among ST types. ST447 and ST451/ST1809, which were the dominant STs in the latter part of our study, had the least amount of AMR genes. In these types, *aac(6')-Ib*, *aac(6')-Ib*, *aadA1*, *ant(3'')-Ia*, and *sul1* were less frequently detected, while *sul*2 and *tet(B)* were the more frequently observed genes. The ST that contained the most abundant AMR genes was ST208/ST1806. This ST possessed the class A extended-spectrum β-lactamase gene *bla*_PER-1_, which is a rare contributor of antimicrobial resistance in *A*. *baumannii*, among others (Table 4).

**Table 4:** Composition of antimicrobial resistance genes according to sequence types.

Gene	Sequence types											
	ST191 (n = 59)	ST357 (n = 7)	ST368 (n = 3)	ST208/ST1806 (n = 5)	ST552 (n = 1)	ST858 (n = 1)	ST369 (n = 3)	ST784 (n = 1)	ST191/ST784 (n = 1)	ST451/ST1809 (n = 11)	ST447 (n = 4)	NA (n = 2)
**Aminoglycoside**												
*aac(3)-Ia*	**36(61.0)**	**0(0)**	3(100)	0(0.0)	0(0.0)	0(0.0)	1(33.3)	0(0.0)	0(0.0)	**0(0.0)**	0(0.0)	2(100)
*aac(6')-Iaf*	0(0.0)	0(0)	**2(66.7)**	0(0.0)	0(0.0)	0(0.0)	0(0.0)	0(0.0)	0(0.0)	0(0.0)	0(0.0)	0(0.0)
*aac(6')-Ib*	**57(96.6)**	7(100)	1(33.3)	5(100)	0(0.0)	1(100)	3(100)	1(100)	1(100)	**0(0.0)**	**1(25.0)**	2(100)
*aac(6')-Ib3*	**57(96.6)**	7(100)	1(33.3)	5(100)	0(0.0)	1(100)	3(100)	1(100)	1(100)	**0(0.0)**	**1(25.0)**	2(100)
*aadA1*	**58(98.3)**	7(100)	1(33.3)	5(100)	0(0.0)	1(100)	3(100)	1(100)	1(100)	**0(0.0)**	**1(25.0)**	2(100)
*aadA1b*	29(50.0)	2(28.6)	3(100)	4(80.0)	0(0.0)	0(0.0)	1(33.3)	1(100)	1(100)	**0(0.0)**	1(25.0)	0(0.0)
*ant(3'')-Ia*	**57(96.6)**	7(100)	1(33.3)	5(100)	0(0.0)	1(100)	3(100)	1(100)	1(100)	**0(0.0)**	**1(25.0)**	2(100)
*aph(3')-Ia*	**0(0.0)**	3(42.9)	0(0.0)	**4(80.0)**	0(0.0)	1(100)	0(0.0)	0(0.0)	0(0.0)	**7(63.6)**	0(0.0)	0(0.0)
*aph(3'')-Ib*	**0(0.0)**	**7(100)**	2(66.7)	**5(100)**	0(0.0)	1(100)	**3(100)**	0(0.0)	0(0.0)	**11(100)**	0(0.0)	0(0.0)
*aph(6)-Id*	**0(0.0)**	**7(100)**	2(66.7)	**5(100)**	0(0.0)	1(100)	**3(100)**	0(0.0)	0(0.0)	**11(100)**	0(0.0)	0(0.0)
*aph(3')-VIb*	**0(0.0)**	0(0)	0(0.0)	**4(80.0)**	0(0.0)	(0.0)	0(0.0)	0(0.0)	0(0.0)	0(0.0)	0(0.0)	0(0.0)
*aph(3')-Vij*	1(1.7)	0(0)	0(0.0)	**4(80.0)**	0(0.0)	0(0.0)	0(0.0)	0(0.0)	0(0.0)	0(0.0)	0(0.0)	0(0.0)
*armA*	**56(94.9)**	7(100)	**1(33.3)**	5(100)	0(0.0)	1(100)	3(100)	1(100)	1(100)	10(90.9)	**1(25.0)**	2(100)
*strA*	**4(6.8)**	**7(100)**	2(66.7)	**5(100)**	0(0.0)	1(100)	2(66.7)	0(0.0)	0(0.0)	**11(100)**	1(25.0)	1(50.0)
**b-lactam**												
*blaADC-25*	59(100)	7(100)	3(100)	5(100)	0(0.0)	1(100)	3(100)	1(100)	1(100)	11(100)	**0(0.0)**	2(100)
*blaOXA-23*	58(98.3)	7(100)	3(100)	5(100)	1(100)	1(100)	3(100)	1(100)	1(100)	11(100)	4(100)	2(100)
*blaOXA-66*	**59(100)**	7(100)	3(100)	5(100)	0(0.0)	1(100)	**1(33.3)**	1(100)	1(100)	11(100)	**1(25.0)**	2(100)
*blaOXA-68*	0(0.0)	0(0)	0(0.0)	0(0.0)	0(0.0)	0(0.0)	0(0.0)	0(0.0)	0(0.0)	0(0.0)	**3(75.0)**	0(0.0)
*blaOXA-82*	0(0.0)	0(0)	0(0.0)	0(0.0)	0(0.0)	0(0.0)	**2(66.7)**	0(0.0)	0(0.0)	0(0.0)	0(0.0)	0(0.0)
*blaOXA-120*	1(1.7)	0(0)	0(0.0)	0(0.0)	1(100)	0(0.0)	0(0.0)	0(0.0)	0(0.0)	0(0.0)	0(0.0)	0(0.0)
*blaPER-1*	0(0.0)	0(0)	0(0.0)	**4(80.0)**	0(0.0)	0(0.0)	0(0.0)	0(0.0)	0(0.0)	0(0.0)	0(0.0)	0(0.0)
*blaTEM-1D*	40(67.8)	5(71.4)	3(100)	3(60)	0(0.0)	0(0.0)	1(33.3)	0(0.0)	0(0.0)	7(63.6)	**0(0.0)**	1(50.0)
**Macrolide**												
*mph(E)*	50(84.7)	7(100)	1(33.3)	5(100)	0(0.0)	1(100)	3(100)	1(100)	1(100)	10(90.9)	**0(0.0)**	1(50.0)
*msr(E)*	53(89.8)	7(100)	**1(33.3)**	5(100)	0(0.0)	1(100)	3(100)	1(100)	1(100)	10(90.9)	**1(25.0)**	2(100)
**Sulphonamide**												
*sul1*	**57(96.6)**	7(100)	1(33.3)	5(100)	0(0.0)	1(100)	3(100)	1(100)	1(100)	**0(0.0)**	**1(25.0)**	2(100)
*sul2*	**0(0.0)**	6(85.7)	2(66.7)	**5(100)**	0(0.0)	1(100)	0(0.0)	0(0.0)	0(0.0)	**11(100)**	**4(100)**	0(0.0)
**Chloramphenicol**												
*catB8*	**56(94.9)**	7(100)	0(0.0)	5(100)	0(0.0)	1(100)	3(100)	1(100)	1(100)	**0(0.0)**	**1(25.0)**	2(100)
**Quinolone**												
*aac(6')-Ib-cr*	**52(88.1)**	7(100)	1(33.3)	5(100)	0(0.0)	1(100)	3(100)	1(100)	1(100)	0(0.0)	**1(25.0)**	2(100)
**Tetracycline**												
*tet(B)*	**0(0.0)**	**7(100)**	2(66.7)	1(20)	0(0.0)	1(100)	2(66.7)	0(0.0)	0(0.0)	**11(100)**	**4(100)**	0(0.0)

Data are expressed as N (%). Values with statistical significance (p-value<0.05) when compared to groups of the rest are expressed in boldface.

Abbreviation: ST, sequence type; NA, not applicable

**Fig 3:**
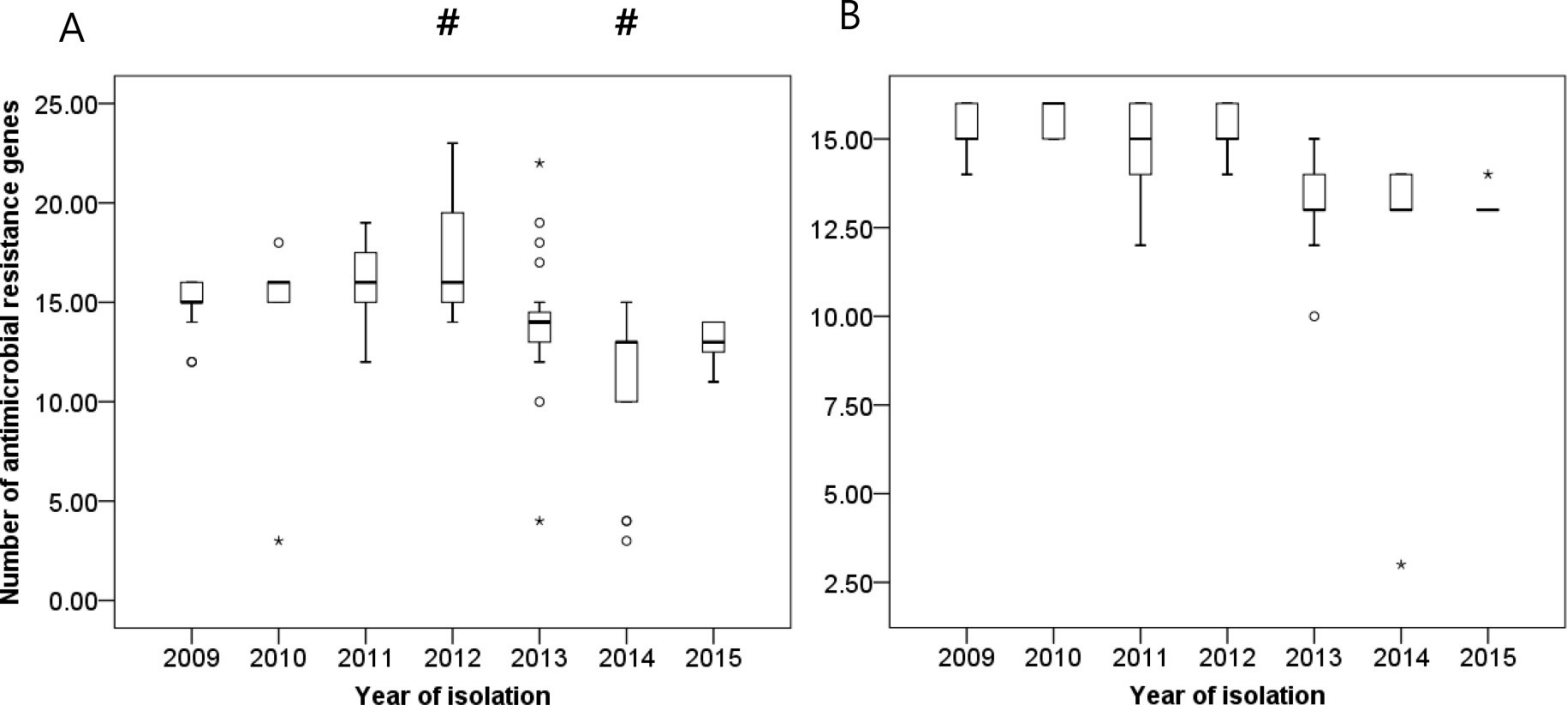
Changes in numbers of antimicrobial resistance genes according to year of isolation. Bar graph represents the medians, percentiles (25^th^ and 75^th^) and 95% confidence intervals: (A) when all sequence types were included and (B) when only sequence type 191 was included. Circle (^○^) and asterisk (*) symbols indicate extreme values. Pound (#) indicates a statistically significant result (p<0.05) as compared to the rest of the year.

The comparison of the total numbers of AMR genes based on the year of isolation showed fluctuating results. The strains isolated in 2012 showed the most abundant AMR genes, while those found in 2014 exhibited the least amount of these genes. In the latter part of the study period, *aac(3)-Ia*, *aac(6')-Ib*, *aac(6')-Ib3*, *aadA1*, *ant(3'')-Ia,blaTEM-1D*, *mph(E)*, *sul1*, and *aac(6')-Ib-cr* were significantly decreased, while *strA* and *tet(B)* were increased. The change was not significant upon separation and chronological comparison of only ST191 (Fig 3A and 3B).

### Differences in virulence genes according to STs and year of isolation

From the literature survey, we compiled a list of 182 potential virulence genes [37]. Of all genes identified, 107 genes were possessed by all of the isolated strains. The most differences among STs were found in the ACICU*_*00075*-*ACICU*_*00087 (immune evasion), *bap*, *csuA*, *casuA/B*, *csuB*, *csuC*, *csuD*, *csuE*, *pgaD* (biofilm formation), and *vgrG1*, *vgrG2*, and *vgrG4* (Type IV protein secretion system) genes (S6 Table). We separated ST191 and searched for epidemiologic differences to find that only *carO*, related to porin, was more frequently detected in the samples originating from the respiratory tract. No statistically significant chronological change was observed (Table 5). The ST that possessed the most abundant virulence genes was ST208/ST1809, which had more ACICU_00075-ACICU_00087, *pgaD*, *bla*_PER-1_, and *hcp* genes compared to the others. The second most common type was ST451/ST1809, which possessed more ACICU_00075-ACICU_00080, ACICU_00086, ACICU_00087, *pgaD*, *pclC1*, and *hcp*. ST552 had the fewest virulence genes and was devoid of *bla*_OXA-24_, ABUW_1966, ACICU_00074-ACICU_00087, *fhaC*, *pgaD*, *bla*_PER-1_, *omp33-36*, *plcC1*, and *hcp*. The ST that had the second fewest virulence genes was ST447 (S6 Table).

**Table 5:** Composition of virulence genes of all sequence types combined and sequence type 191 according to isolation date or source of bacteremia.

Genes	ST191						ALL		
	Source of bacteremia			Isolated year			Isolated year		
	Respiratory tract(n = 46)	Other(n = 13)	p-value	2009-2012(n = 37)	2013-2015(n = 22)	p-value	2009-2012(n = 37)	2009-2012(n = 37)	p-value
**Outer membrane vesicle**									
A1S_0009	46(10)	13(100)	1	37(100)	22(100)	1	54(100)	44(100)	1
A1S_0116	46(10)	13(100)	1	37(100)	22(100)	1	50(92.6)	39(88.6)	0.37
A1S_1180	46(10)	13(100)	1	37(100)	22(100)	1	54(100)	44(100)	1
A1S_1321	45(97.8)	13(100)	0.78	36(97.3)	22(100)	0.63	53(98.1)	44(100)	0.55
A1S_1386	46(10)	13(100)	1	37(100)	22(100)	1	54(100)	44(100)	1
A1S_1510	46(10)	13(100)	1	37(100)	22(100)	1	53(98.1)	44(100)	0.55
A1S_1921	46(10)	13(100)	1	37(100)	22(100)	1	54(100)	44(100)	1
A1S_2470	46(10)	13(100)	1	37(100)	22(100)	1	54(100)	44(100)	1
A1S_2525	46(10)	13(100)	1	37(100)	22(100)	1	54(100)	44(100)	1
A1S_3143	46(10)	13(100)	1	37(100)	22(100)	1	54(100)	44(100)	1
A1S_3175	46(10)	13(100)	1	37(100)	22(100)	1	54(100)	44(100)	1
A1S_3411	46(10)	13(100)	1	37(100)	22(100)	1	54(100)	44(100)	1
*ACV72173*.*1*	43(93.5)	13(100)	0.47	34(91.9)	22(100)	0.24	**46(85.2)**	**43(97.7)**	**0.03**
*ACV72174*.*1*	43(93.5)	13(100)	0.47	34(91.9)	22(100)	0.24	47(87.0)	43(97.7)	0.06
*ADB23465*.*1*	43(93.5)	13(100)	0.47	34(91.9)	21(95.5)	0.52	47(87.0)	43(97.7)	0.06
*ADB23466*.*1*	39(84.8)	12(92.3)	0.43	31(83.8)	20(90.9)	0.36	40(	39(	
*ADB23467*.*1*	43(93.5)	12(92.3)	0.64	34(91.9)	21(95.5)	0.52	47(87.0)	42(95.5)	0.14
*ADB23468*.*1*	43(93.5)	13(100)	0.47	34(91.9)	22(100)	0.24	47(87.0)	43(97.7)	0.06
*ADB23470*.*1*	43(93.5)	13(100)	0.47	34(91.9)	22(100)	0.24	47(87.0)	43(97.7)	0.06
*ADB23471*.*1*	40(87.0)	13(100)	0.21	33(89.2)	20(90.9)	0.60	45(83.3)	41(93.2)	0.126
*ADB23472*.*1*	37(80.4)	12(92.3)	0.29	28(75.7)	21(95.5)	0.07	**41(75.9)**	**40(90.9)**	**0.04**
*ADB23473*.*1*	42(91.3)	13(100)	0.36	33(89.2)	22(100)	0.15	**46(85.2)**	**43(97.7)**	**0.03**
*GADB23474*.*1*	43(93.5)	13(100)	0.47	34(91.9)	22(100)	0.24	**46(85.2)**	**43(97.7)**	**0.03**
*GADB23475*.*1*	43(93.5)	13(100)	0.47	34(91.9)	22(100)	0.24	47(87.0)	43(97.7)	0.06
*bla*_OXA-24_	0(0.0)	0(0.0)	1	0(0.0)	0(0.0)	1	0(0)	0(0)	1
**Antibiotic resistance**									
ABUW_1156	46(10)	13(100)	1	37(100)	22(100)	1	53(98.1)	40(90.9)	0.12
ABUW_1499	43(93.5)	13(100)	0.47	36(97.3)	20(90.9)	0.31	45(83.3)	38(86.4)	0.45
ABUW_1520	41(89.2)	13(100)	0.27	35(94.6)	19(86.4)	0.26	**45(83.3)**	**26(59.1)**	**<0.01**
ABUW_1645	46(10)	13(100)	1	37(100)	22(100)	1	54(100)	44(100)	1
ABUW_1672	46(10)	13(100)	1	37(100)	22(100)	1	54(100)	44(100)	1
ABUW_1673	46(10)	13(100)	1	37(100)	22(100)	1	53(98.1)	44(100)	0.55
ABUW_1692	45(97.8)	13(100)	0.78	36(97.3)	22(100)	0.63	53(98.1)	44(100)	0.55
ABUW_1755	46(10)	13(100)	1	37(100)	22(100)	1	54(100)	44(100)	1
ABUW_1768	46(10)	13(100)	1	37(100)	22(100)	1	54(100)	44(100)	1
ABUW_1849	46(10)	13(100)	1	37(100)	22(100)	1	54(100)	44(100)	1
ABUW_1851	46(10)	13(100)	1	37(100)	22(100)	1	54(100)	44(100)	1
ABUW_1966	46(10)	13(100)	1	37(100)	22(100)	1	0(0)	0(0)	1
ABUW_2074	46(10)	13(100)	1	37(100)	22(100)	1	54(100)	44(100)	1
ABUW_2550	45(97.8)	13(100)	0.78	36(97.3)	22(100)	0.63	53(98.1)	44(100)	0.55
**Aromatic hydrocarbon metabolism**									
*ABUW_2090*	46(10)	13(100)	1	37(100)	22(100)	1	54(100)	44(100)	1
*ABUW_2123*	46(10)	13(100)	1	37(100)	22(100)	1	54(100)	44(100)	1
*ABUW_2236*	46(10)	13(100)	1	37(100)	22(100)	1	54(100)	44(100)	1
*ABUW_2349*	46(10)	13(100)	1	37(100)	22(100)	1	54(100)	44(100)	1
*ABUW_2370*	46(10)	13(100)	1	37(100)	22(100)	1	54(100)	44(100)	1
*ABUW_2374*	46(10)	13(100)	1	37(100)	22(100)	1	54(100)	44(100)	1
*benP1*	46(10)	13(100)	1	37(100)	22(100)	1	54(100)	44(100)	1
*paaI1*	46(10)	13(100)	1	37(100)	22(100)	1	54(100)	44(100)	1
*paaY*	46(10)	13(100)	1	37(100)	22(100)	1	54(100)	44(100)	1
*pcaC*	46(10)	13(100)	1	37(100)	22(100)	1	54(100)	44(100)	1
*pcaD1*	46(10)	13(100)	1	37(100)	22(100)	1	54(100)	44(100)	1
*pcaU*	46(10)	13(100)	1	37(100)	22(100)	1	54(100)	44(100)	1
**Transcriptional regulation**									
ABUW_2520	46(10)	13(100)	1	37(100)	22(100)	1	54(100)	44(100)	1
ABUW_2544	45(97.8)	13(100)	0.78	36(97.3)	22(100)	0.63	52(96.3)	44(100)	0.30
**Immune evasion**									
ACICU_00074	46(10)	13(100)	1	37(100)	22(100)	1	54(100)	44(100)	1
ACICU_00075	0(0.0)	0(0.0)	1	0(0.0)	0(0.0)	1	**4(7.4)**	**12(27.3)**	**<0.01**
ACICU_00076	0(0.0)	0(0.0)	1	0(0.0)	0(0.0)	1	**4(7.4)**	**12(27.3)**	**<0.01**
ACICU_00077	0(0.0)	0(0.0)	1	0(0.0)	0(0.0)	1	**4(7.4)**	**12(27.3)**	**<0.01**
ACICU_00078	0(0.0)	0(0.0)	1	0(0.0)	0(0.0)	1	**4(7.4)**	**12(27.3)**	**<0.01**
ACICU_00079	0(0.0)	0(0.0)	1	0(0.0)	0(0.0)	1	**4(7.4)**	**12(27.3)**	**<0.01**
ACICU_00080	0(0.0)	0(0.0)	1	0(0.0)	0(0.0)	1	**4(7.4)**	**12(27.3)**	**<0.01**
ACICU_00081	0(0.0)	0(0.0)	1	0(0.0)	0(0.0)	1	4(7.4)	1(2.3)	0.25
ACICU_00082	0(0.0)	0(0.0)	1	0(0.0)	0(0.0)	1	4(7.4)	1(2.3)	0.25
ACICU_00083	0(0.0)	0(0.0)	1	0(0.0)	0(0.0)	1	4(7.4)	1(2.3)	0.25
ACICU_00084	0(0.0)	0(0.0)	1	0(0.0)	0(0.0)	1	4(7.4)	1(2.3)	0.25
ACICU_00085	0(0.0)	0(0.0)	1	0(0.0)	0(0.0)	1	4(7.4)	1(2.3)	0.25
ACICU_00086	0(0.0)	0(0.0)	1	0(0.0)	0(0.0)	1	**7(13.0)**	**15(34.1)**	**0.01**
ACICU_00087	0(0.0)	0(0.0)	1	0(0.0)	0(0.0)	1	**7(13.0)**	**15(34.1)**	**0.01**
ACICU_00088	46(10)	13(100)	1	37(100)	22(100)	1	54(100)	44(100)	1
ACICU_00089	46(10)	13(100)	1	37(100)	22(100)	1	54(100)	44(100)	1
ACICU_00091	46(10)	13(100)	1	37(100)	22(100)	1	54(100)	44(100)	1
ACICU_00092	46(10)	13(100)	1	37(100)	22(100)	1	54(100)	44(100)	1
*lpsB*	46(10)	13(100)	1	37(100)	22(100)	1	54(100)	44(100)	1
*lptE*	46(10)	13(100)	1	37(100)	22(100)	1	54(100)	44(100)	1
*lpxA*	46(10)	13(100)	1	37(100)	22(100)	1	54(100)	44(100)	1
*lpxB*	46(10)	13(100)	1	37(100)	22(100)	1	54(100)	44(100)	1
*lpxC*	46(10)	13(100)	1	37(100)	22(100)	1	54(100)	44(100)	1
*lpxD*	46(10)	13(100)	1	37(100)	22(100)	1	54(100)	44(100)	1
*lpxL*	46(10)	13(100)	1	37(100)	22(100)	1	54(100)	44(100)	1
*lpxM*	46(10)	13(100)	1	37(100)	22(100)	1	54(100)	44(100)	1
*eps*	46(10)	13(100)	1	37(100)	22(100)	1	**43(79.6)**	**42(95.5)**	**0.02**
*pgi*	46(10)	13(100)	1	37(100)	22(100)	1	54(100)	44(100)	1
*ptk*	46(10)	13(100)	1	37(100)	22(100)	1	54(100)	44(100)	1
*ptp*	46(10)	13(100)	1	37(100)	22(100)	1	54(100)	44(100)	1
**Regulation**									
*abaI*	46(10)	13(100)	1	37(100)	22(100)	1	50(92.6)	39(88.6)	0.37
*abaR*	43(93.5)	11(84.6)	0.30	36(97.3)	18(81.8)	0.06	49(90.7)	35(79.5)	0.10
*bfmR*	46(10)	13(100)	1	37(100)	22(100)	1	54(100)	44(100)	1
*bfmS*	46(10)	13(100)	1	37(100)	22(100)	1	54(100)	44(100)	1
**Killing of host cells**									
*abeD*	46(10)	13(100)	1	37(100)	22(100)	1	54(100)	44(100)	1
*envZ*	46(10)	13(100)	1	37(100)	22(100)	1	54(100)	44(100)	1
*fhaB*	45(97.8)	13(100)	0.78	36(97.3)	22(100)	0.63	**52(96.3)**	**37(84.1)**	**0.04**
*fhaC*	0(0.0)	0(0.0)	1	0(0.0)	0(0.0)	1	1(1.9)	0(0)	0.55
**Biofilm formation**									
*adeF*	46(10)	13(100)	1	37(100)	22(100)	1	54(100)	44(100)	1
*adeG*	46(10)	13(100)	1	37(100)	22(100)	1	54(100)	44(100)	1
*bap*	46(10)	13(100)	1	37(100)	22(100)	1	53(98.1)	40(90.9)	0.12
*csuA*	44(95.7)	13(100)	0.61	37(100)	20(90.9)	0.14	47(87.0)	36(81.8)	0.33
*csuA/B*	44(95.7)	13(100)	0.61	37(100)	20(90.9)	0.14	47(87.0)	35(79.5)	0.23
*csuB*	44(95.7)	13(100)	0.61	37(100)	20(90.9)	0.14	47(87.0)	35(79.5)	0.23
*csuC*	44(95.7)	13(100)	0.61	37(100)	20(90.9)	0.14	47(87.0)	36(81.8)	0.33
*csuD*	44(95.7)	13(100)	0.61	37(100)	20(90.9)	0.14	47(87.0)	36(81.8)	0.33
*csuE*	44(95.7)	13(100)	0.61	36(97.3)	20(90.9)	0.31	46(85.2)	36(81.8)	0.43
*pgaA*	46(10)	13(100)	1	37(100)	22(100)	1	53(98.1)	44(100)	0.55
*pgaB*	46(10)	13(100)	1	37(100)	22(100)	1	54(100)	44(100)	1
*pgaC*	46(10)	13(100)	1	37(100)	22(100)	1	**54(100)**	**40(90.9)**	**0.04**
*pgaD*	0(0.0)	0(0.0)	1	0(0.0)	0(0.0)	1	15(27.8)	19(43.2)	0.14
*adeH*	46(10)	13(100)	1	37(100)	22(100)	1	54(100)	44(100)	1
**Antibiotic resisance**									
*adeI*	46(10)	13(100)	1	37(100)	22(100)	1	54(100)	44(100)	1
*adeJ*	46(10)	13(100)	1	37(100)	22(100)	1	54(100)	44(100)	1
*adeK*	46(10)	13(100)	1	37(100)	22(100)	1	54(100)	44(100)	1
*bla*_PER-1_	0(0.0)	0(0.0)	1	0(0.0)	0(0.0)	1	3(5.6)	1(2.3)	0.39
**Transcriptional regulation**									
*alkR*	46(10)	13(100)	1	37(100)	22(100)	1	54(100)	44(100)	1
*gigA*	46(10)	13(100)	1	37(100)	22(100)	1	54(100)	44(100)	1
*gigB*	46(10)	13(100)	1	37(100)	22(100)	1	54(100)	44(100)	1
*gigC*	46(10)	13(100)	1	37(100)	22(100)	1	54(100)	44(100)	1
*soxR*	45(97.8)	13(100)	0.78	36(97.3)	22(100)	0.63	53(98.1)	44(100)	0.55
**Iron uptake**									
*barA*	46(10)	13(100)	1	37(100)	22(100)	1	54(100)	44(100)	1
*barB*	46(10)	13(100)	1	37(100)	22(100)	1	54(100)	44(100)	1
*basA*	46(10)	13(100)	1	37(100)	22(100)	1	54(100)	44(100)	1
*basB*	46(10)	13(100)	1	37(100)	22(100)	1	54(100)	44(100)	1
*basC*	46(10)	13(100)	1	37(100)	22(100)	1	54(100)	44(100)	1
*basD*	46(10)	13(100)	1	37(100)	22(100)	1	54(100)	44(100)	1
*basF*	46(10)	13(100)	1	37(100)	22(100)	1	54(100)	44(100)	1
*basG*	46(10)	13(100)	1	37(100)	22(100)	1	54(100)	44(100)	1
*basH*	46(10)	13(100)	1	37(100)	22(100)	1	54(100)	44(100)	1
*basI*	46(10)	13(100)	1	37(100)	22(100)	1	54(100)	44(100)	1
*basJ*	46(10)	13(100)	1	37(100)	22(100)	1	54(100)	44(100)	1
*bauA*	46(10)	13(100)	1	37(100)	22(100)	1	**54(100)**	**40(90.9)**	**0.04**
*bauB*	46(10)	13(100)	1	37(100)	22(100)	1	54(100)	44(100)	1
*bauC*	46(10)	13(100)	1	37(100)	22(100)	1	54(100)	44(100)	1
*bauD*	46(10)	13(100)	1	37(100)	22(100)	1	54(100)	44(100)	1
*bauE*	46(10)	13(100)	1	37(100)	22(100)	1	54(100)	44(100)	1
*bauF*	46(10)	13(100)	1	37(100)	22(100)	1	54(100)	44(100)	1
*nfuA*	46(10)	13(100)	1	37(100)	22(100)	1	54(100)	44(100)	1
**Porin**									
*carO*	**46(100)**	**11(84.6)**	**0.046**	37(100)	20(90.9)	0.14	**54(100)**	**40(90.9)**	**0.04**
*omp22*	46(10)	13(100)	1	37(100)	22(100)	1	54(100)	44(100)	1
*omp33-36*	0(0.0)	0(0.0)	1	37(100)	22(100)	1	0	0	
*ompR*	46(10)	13(100)	1	37(100)	22(100)	1	54(100)	44(100)	1
*orpD*	46(10)	13(100)	1	37(100)	22(100)	1	54(100)	44(100)	1
**Serun resistance, invasion**									
*cipA*	46(10)	13(100)	1	37(100)	22(100)	1	54(100)	44(100)	1
*cobA*	46(10)	13(100)	1	37(100)	22(100)	1	54(100)	44(100)	1
*pbpG*	46(10)	13(100)	1	37(100)	22(100)	1	54(100)	44(100)	1
*surA1*	46(10)	13(100)	1	37(100)	22(100)	1	54(100)	44(100)	1
*tuf*	46(10)	13(100)	1	36(97.3)	22(100)	0.63	53(98.1)	44(100)	0.55
*typA*	46(10)	13(100)	1	37(100)	22(100)	1			
**Enzyme**									
*plcC1*	0(0.0)	0(0.0)	1	0(0.0)	0(0.0)	1	8(14.8)	14(31.8)	0.05
*plcC2*	46(10)	13(100)	1	37(100)	22(100)	1	54(100)	44(100)	1
*plcD*	46(10)	13(100)	1	37(100)	22(100)	1	54(100)	44(100)	1
*pldA*	46(10)	13(100)	1	37(100)	22(100)	1	54(100)	44(100)	1
**Cysteine metabolism**									
*cysD*	46(10)	13(100)	1				54(100)	44(100)	1
*cysE*	46(10)	13(100)	1	37(100)	22(100)	1	54(100)	44(100)	1
*cysH*	46(10)	13(100)	1	37(100)	22(100)	1	54(100)	44(100)	1
*cysI*	46(10)	13(100)	1	37(100)	22(100)	1	54(100)	44(100)	1
*cysN*	46(10)	13(100)	1	37(100)	22(100)	1	54(100)	44(100)	1
*cysQ*	46(10)	13(100)	1	37(100)	22(100)	1	54(100)	44(100)	1
*sulP*	46(10)	13(100)	1	37(100)	22(100)	1			
**Siderophore biosynthesis**									
*entA*	46(100)	13(100)	1	37(100)	22(100)	1	54(100)	42(95.5)	0.20
**Neutrophil influx**									
*gacS*	46(10)	13(100)	1				54(100)	44(100)	1
*paaA*	45(97.8)	13(100)	0.78	36(97.3)	22(100)	0.63	53(98.1)	44(100)	0.55
**Type II protein secretion system**									
*gspD*	46(10)	13(100)	1	37(100)	22(100)	1	54(100)	44(100)	1
**Type VI protein secretion system**									
*vgrG1*	46(10)	13(100)	1	37(100)	22(100)	1	39(72.2)	27(61.4)	0.29
*vgrG2*	46(10)	13(100)	1	37(100)	22(100)	1	**52(96.3)**	**33(75.0)**	**<0.01**
*vgrG3*	46(10)	13(100)	1	37(100)	22(100)	1	53(98.1)	44(100)	0.55
*vgrG4*	46(10)	13(100)	1	37(100)	22(100)	1	40(74.1)	27(61.4)	0.20
*hcp*	0(0.0)	0(0.0)	1	0(0.0)	0(0.0)	1	12(22.2)	15(34.1)	0.14
**Type V protein secretion system**									
*ata*	41(89.1)	12(92.3)	0.60	31(83.8)	22(100)	0.08	42(77.8)	39(88.6)	0.13
**Stress response genes**									
*kef*	46(10)	13(100)	1	37(100)	22(100)	1	54(100)	44(100)	1
*kefF*	45(97.8)	13(100)	0.78	36(97.3)	22(100)	0.63	54(100)	44(100)	1
*mscS*	46(10)	13(100)	1	37(100)	22(100)	1	54(100)	44(100)	1
*ostB*	46(10)	13(100)	1	37(100)	22(100)	1	54(100)	44(100)	1
*recA*	46(10)	13(100)	1	37(100)	22(100)	1	54(100)	44(100)	1
*resP*	46(10)	13(100)	1	37(100)	22(100)	1	54(100)	44(100)	1
*trkH*	46(10)	13(100)	1	37(100)	22(100)	1	54(100)	44(100)	1
*upsA*	46(10)	13(100)	1	37(100)	22(100)	1	54(100)	44(100)	1
*uspA*	0(0.0)	0(0.0)	1	0(0.0)	0(0.0)	1	0(0)	0(0)	1
*uvrD*	0(0.0)	0(0.0)	1	0(0.0)	0(0.0)	1	0(0)	0(0)	1
**Manganese acquisition system**									
*mumC*	45(97.8)	13(100)	0.78	36(97.3)	22(100)	0.63	52(96.3)	44(100)	0.30
*mumT*	45(97.8)	13(100)	0.78	36(97.3)	22(100)	0.63	52(96.3)	44(100)	0.30
**Adherence**									
*ompA*	46(10)	13(100)	1	37(100)	22(100)	1	54(100)	44(100)	1
**Micronutrient acquisition**									
*znuA*	46(10)	13(100)	1	37(100)	22(100)	1	54(100)	44(100)	1
*znuB*	46(10)	13(100)	1	37(100)	22(100)	1	54(100)	44(100)	1
*znuC*	46(10)	13(100)	1	37(100)	22(100)	1	54(100)	44(100)	1
*zur*	46(10)	13(100)	1	37(100)	22(100)	1	54(100)	44(100)	1

Data are expressed as N (%). Values with statistical significance (p-value<0.05) are expressed in boldface.

**Fig 4:**
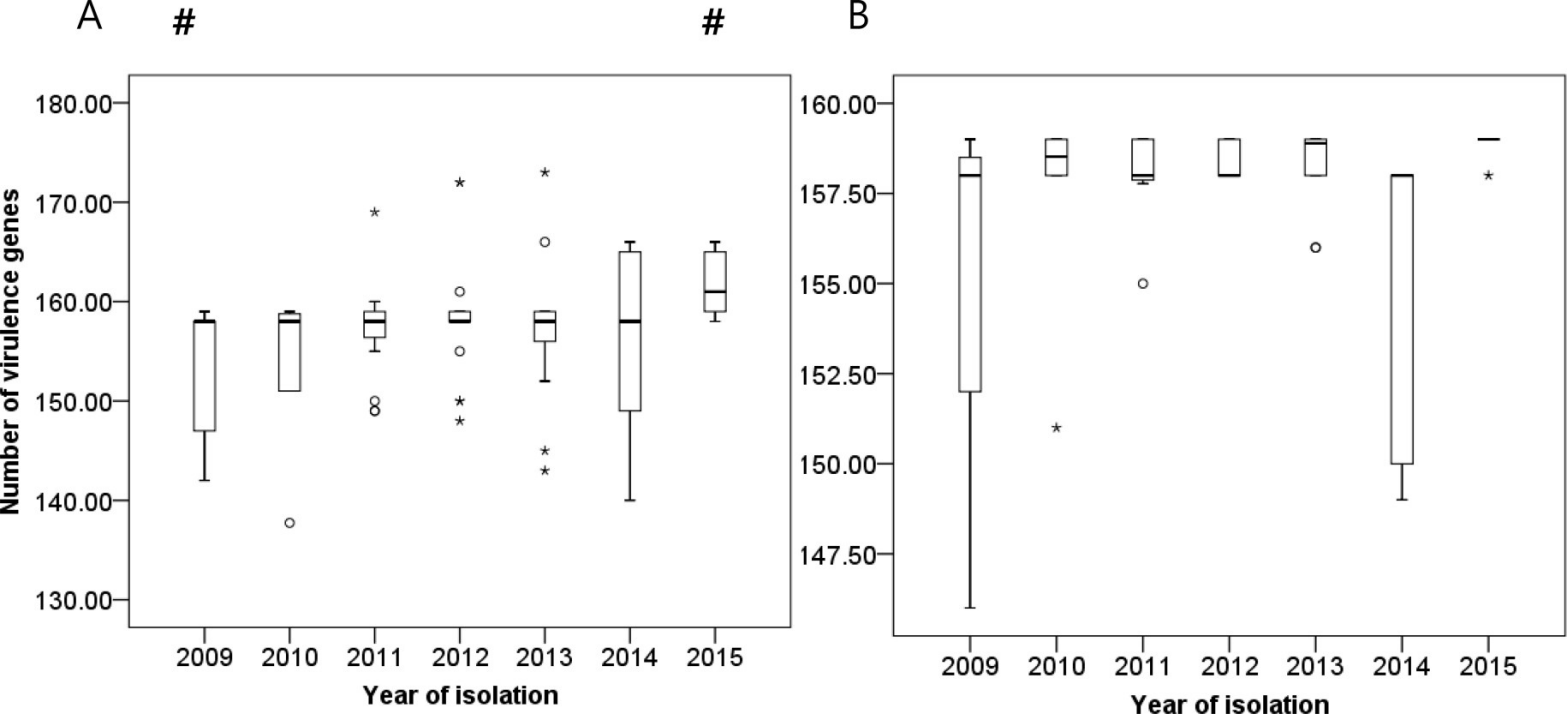
Changes in numbers of virulence genes according to year of isolation. Bar graph represents medians, percentiles (25^th^ and 75^th^), and 95% confidence intervals: (A) when all sequence types were included and (B) when only sequence type 191 was included. Circle (^○^) and asterisk (*) symbols indicate extreme values. Pound (#) indicates a statistically significant result (p<0.05) as compared to the rest of the year.

The number of virulence genes was compared according to the year of isolation. The number increased as research period elapsed. The strains isolated in the year 2009 carried fewer virulence genes, while those isolated in 2015 exhibited more virulence genes compared to the rest. We singled out ST191 and observed statistically insignificant results (Fig 4A and 4B).

### Difference in IS*Aba1* and carbapenemase-encoding transposons on CRAB

IS*Aba1* belongs to the IS*4* family and has been detected upstream of the *ampC*, *bla*_OXA-23_, *bla*_OXA-27_, and *sul*2 antibiotic resistance genes in *Acinetobacter* species [34]. Insertion sites of IS*Aba1* were different for each strain, and no common insertion sites were conserved throughout all strains. Insertion sites of IS*Aba1* that were shared by more than 70% of strains were designated as IS*1, IS*2, IS*3, IS*4, IS*5, IS*6, and IS*7. The compositions of IS*1 through IS*7 differed between STs (S7 Table). ST447 contained fewer insertion sequences compared to the rest, while ST191 carried comparatively more insertion sequences; these incidences remained constant throughout the study period for each insertion sequence (S1 Fig). We could detect common *bla*_OXA-23_-ΔATPase modules from all isolates with the exception of ABAY11010 (ST369) and ABAY12016 (ST208/ST1806), which implied that Tn*2006*, Tn*2008*, or Tn*2009* was widespread among these strains. However, although antimicrobial resistance (AMR) genes were predicted using three sets of contig sequences, which include assemblies obtained by CLC Genomics Workbench (whole-genome assembly), by plasmid SPAdes, and by plasmid profiler-based process, these cannot determine the exact location of AMR genes. ABAY11010 and ABAY12016 originated in the respiratory tract, and had larger number of virulence and AMR genes than average (virulence genes, 160 and 161 copies each; AMR genes, 16 and 23 copies each). Both strains were isolated at different places (MICU D and MICU C each) and times (2011 and 2012 each).

### Resistance islands

AbaR1 is a commonly reported sequence from strains isolated in South Korea [38,39]. We searched contig sequences using the 87.7-kb AbaR1 sequence from the strain AYE (CU459141.1) [40] as a query, and found 70 strains to carry a very short AbaR-like sequence. These were only 10,641 bp long after including the disrupted ATPase gene (*comM’*) at both ends. AbaR-like islands were similar to the 14.5-kb long Tn*6021* that is devoid of the multiple antibiotic resistance region found in ATCC 17978 (GenBank CP00521.1). When read mapping on the complete AbaR-like sequence extracted from ABAY09001 (GenBank QHGS01000008.1; 56,153–66,793 bp) was applied, all strains except ABAY13016, ABAY14002, ABAY14004, and ABAY14008 (all categorised as ST447) possessed AbaR-like islands.

Also, 75 ABAY strains harbor class I integron carrying *cat2*, *aadA1*, *sul1* and *armA*. It has been reported that two consecutive copies of integrons are located within a putative resistance island, but read mapping-based analysis (this study) could not reveal how integrons are situated in ABAY chromosomes. The gene organization is similar with class I integron identified from NCGM 237 [41].

### Reconstruction of plasmid sequence

Assembly-based methods produced putative plasmidic contigs from all 98 isolates: average total lengths of plasmidic contigs predicted by plasmiSPAdes and plasmid profiler-based unicycler were 129,195.1 bp and 141,235 bp, respectively. Standard deviations of their total lengths were 136,737.5 bp and 71,471.1 bp, respectively. The latter method, which relies on graph-based assembly followed by circularization, identified 115 complete plasmid sequences along with linear and complex type assemblies. We clustered plasmid sequences into groups based on sequence similarity and length, which resulted in five groups ranging from 110,967 bp (group A) to 2,278 bp (group E) (S8 Table). To estimate the presence of all five groups of plasmids across strains, sequencing reads were re-mapped on the representative plasmid sequences (S9 Table). Unexpectedly, we could not find any antimicrobial resistance genes from the five representative plasmid sequences. However, unresolved plasmids in the plasmid profiler-based unicycler assemblies might contain not-yet-identified ones that harbor multiple AMR determinants.

We identified IS*Aba1*, which is known to promote expression of downstream genes, from four complete plasmids in group B. However, no *bla*_OXA-23_ was found in their vicinity or elsewhere in the complete plasmid sequences.

## Discussion

Since the 1970s, there has been a progressive increase in the antimicrobial resistance for the majority of *A*. *baumannii* strains, which were otherwise sensitive to the commonly used antibiotics [42]. By 2007, up to 70% of isolates in certain settings had developed multidrug resistance including resistance to carbapenems, which were once considered as the mainstay against multidrug-resistant *A*. *baumannii* infections [43]. In our study, ST191 was the dominant ST during the 7 years of our study and showed stable genomic variations. However, when other STs were combined, a tendency toward increasing virulence genes was observed without additional changes in antimicrobial resistance of CRAB in restricted hospital environments.

ST191 is a predominant strain isolated in South Korea [44] known for expressing *bla*_**OXA-23**_, which is responsible for the high rate of carbapenem resistance. The trait that allows this strain to emerge as a highly successful nosocomial pathogen through frequent modification of genetic components was also manifested in our study. ST191 had higher insertion sequences compared to the other types, indicating the frequent recombination and gene rearrangement events. Also, only 60% of strains involved in our study had closed related strains identified in previous studies. However, no cumulative change in AMR genes and virulence genes was observed over time. The only gene that showed increased prevalence was *strA*. This gene is related to aminoglycoside resistance and is frequently found in AbaR-like islands along with other genes such as *tetA*(B), *tetR*(B), *CR2*, *strB*, and *orf4b* [45]. We observed no simultaneous increase in other AMR genes in this study.

It is worth noting that heterogenous groups of ST were isolated simultaneously. ST208/ST1806 was the sequence type with the most abundant AMR genes; *bla*_**PER-1**_ was found only in this sequence type. Although it is a relatively uncommon gene for *A*. *baumannii*, *bla*_**PER-1**_ is frequently reported as being implicated in various virulence mechanisms [46,47]. In our study, this strain was isolated mostly in 2012, and additional spread of this gene was not noted. ST447, which was categorised into international clone 1 (IC I), was isolated in the latter part of our study period. ST447 possessed fewer AMR genes. Since this strain was more frequently isolated from patients with shorter hospital stays, it was introduced from the outside rather than being an indigenous strain. This type was equipped with β-lactam resistance genes without any other resistance genes. As with other strains, ST447 also had *bla*_**OXA-23**_-ΔATPase modules, indicating that it carries Tn*2006*, Tn*2008*, or Tn*2009*. While Tn*2006* was mostly transferred by mobile elements, the spread of Tn*2008* and Tn*2009* was entirely dependent on the clonal dissemination of the bacterial host [44]. In light of ST447 containing all of these transposons, this type might have been under selective antibiotic pressure for a long period of time. It is possible that the previously susceptible strain may have gained genes only for carbapenem resistance during the study period, but the widespread use of carbapenems may have contributed to the emergence of this type. The density of virulence genes was still low, and this type of strain had relatively lower insertion sequences, which are commonly found in other STs. This defies the concern posed by a previous study in South Korea which reported the emergence of a new strain that would provoke the dissemination of more antibacterial resistance [48].

Despite sporadic studies suggesting correlation between the number of virulence genes with organisms’ phenotypic differences [16], the significance of number of virulence genes in association with clinical outcomes and epidemiology has not been investigated. Therefore, the increased number of virulence genes over time shown in our study demands immediate attention. Although previous studies suggested certain associations between antimicrobial resistance and virulence [49], our study demonstrated that linear correlation between the two did not exist. It is worth noting that more than half of the highly significant virulence genes were possessed by all of these carbapenem-resistant strains. A slight increase in the number of virulence genes was reported in 2015, probably due to the emergence of ST451/ST1809. ST451/ST1809 possessed higher number of virulence genes than average (165 [159–166] vs. 158[138–173], p<0.001). A gene specifically related to the functioning of porin (*carO)* [50] had decreased, which may be attributed to enhanced antimicrobial resistance. However, genes that were previously considered important in forming biofilms and iron utilization (*bauA* and *pgaC*) [49,51] decreased in number. The increased genes were ACICU*_*00075*-*ACICU*_*00080, ACICU_00086, and ACICU_00087, which are presumably associated with immune evasion [52]. Although further evaluation is warranted regarding the authentic effects of these putative virulence genes, the gradual accumulation of virulence genes that are transmitted by horizontal transfer emphasises the need for more intensive infection control strategies to prevent re-infections of CRAB.

We failed to observe any significant differences in patient outcomes according to STs. As more than 80% of patients died within 28 days of the first isolation of the organism, it is possible that the patients were in serious condition and hence died before the analysis reached a conclusive stage. However, previous studies also suggested that phenotypical characteristics are not in accordance with genotypical results. It is possible that individual virulence factors may not be important for *A*. *baumannii* virulence in human hosts [53], and the same virulence factor may play different roles in different habitats [54]. The expression of virulence-associated genes could be under different regulation in pathogenic and non-pathogenic species [55].

We found no discrepancies between phenotypic and genotypic results in terms of carbapenem resistance. This observation confirmed the results of a previous study, in which WGS accurately identified resistance to β-lactam antibiotics for gram-negative bacterial pathogens [56]. Whether this observation holds true for other types of antimicrobial agents warrants further studies.

The current study had a few limitations. First, only patients who were admitted to ICUs were involved, limiting further epidemiological investigation. Second, the relationships between strains, such as evolutionary linkages, were not determined. Third, detailed comparative genomics to verify mechanisms of acquisition of virulence or AMR genes acquisition were not analysed.

In conclusion, this study confirms the absence of accumulation of antimicrobial resistance determinants while the number of virulence genes increases in CRAB. Genetic transfer between subtypes cannot be ruled out; hence, it is important to be cautious about re-infections.

## Supporting information

S1 Table List of virulence factor genes. (XLSX)

S2 Table Sequencing and assembly statistics. (XLSX)

S3 Table Insertion sites of ISAba1 relative to the chromosome of KBN10P02143. (XLSX)

S4 Table Phenotypic antimicrobial susceptibility of carbapenem resistant *A*.*baumanii*. (DOCX)

S5 Table Pairwise SNP distances within and between clades. (DOCX)

S6 Table Composition of virulence genes according to sequence types. (DOCX)

S7 Table Differences in distribution of insertion sequences according to sequence types. (DOCX)

S8 Table Summary of complete plasmids identified from plasmid profiler-based assembly. (XLSX)

S9 Table Distribution of plasmids across 98 strains as identified using read mapping on the five respresentative plasmid sequences. (XLSX)

S1 Fig Cumulative prevalence of each insertion sequences of ST191. Bar graph represents individual and cumulative number of each sequence types according to year of isolation.
(TIF)
