# Supplementary material for: Using comparative genomics to understand molecular features of carbapenem-resistant Acinetobacter baumannii from South Korea causing invasive infections and their clinical implications
Source: PLoS One. 2020 Feb 21;15(2):e0229416. doi: 10.1371/journal.pone.0229416 (PMC7034955; doi:10.1371/journal.pone.0229416)
Supplement: S4 Table — (DOCX) [file pone.0229416.s004.docx]

**Supplementary Table S4. Phenotypic antimicrobial susceptibility of carbapenem resistant *A.baumanii***

Data are expressed as N/N (%).Denominators indicate number of patients whose data available in each group. Abbreviation: GEN, gentamycin; AMK, amikacin; IMP, imipenem; TZP, piperacillin-tazobactam; CAZ, cefazidime; FEP, cefepime; CIP, ciprofloxacin; LVX, levofloxacin; CST, colistin; TET, tetracycline; NA, not available

Data with discrepant results were expressed.

| **Antibiotics** | Number (%) of isolates collected in this study susceptible each antimicrobial agent | | | | | | | | | | | | |
| --- | --- | --- | --- | --- | --- | --- | --- | --- | --- | --- | --- | --- | --- |
|  | ST191 (n=59) | ST357  (n=7) | ST368 (n=3) | ST208/ST1806 (n=5) | ST552 (n=1) | ST858 (n=1) | ST369  (n=3) | ST784 (n=1) | ST191/ST784 (n=1) | ST451/ST1809 (n=11) | | ST447 (n=4) | NA (n=2) |
| **GEN** | 3/57 (5.3) | 0/7(0.0) | 0/3 (0.0) | 0/5 (0.0) | 1/1 (100) | 0/1 (0.0) | 0/3 (0.0) | 0/1 (0.0) | 0/1 (0.0) | 0/11 (0.0) | | 3/4 (75.0) | 0/2(0.0) |
| **AMK** | 6/59 (10.2) | 2/7(28.6) | 0/3 (0.0) | 1/5 (20.0) | 1/1 (100) | 0/1 (0.0) | 0/3 (0.0) | 0/1 (0.0) | 0/1 (0.0) | | 0/11 (0.0) | 3/4 (75.0) | 0/2(0.0) |
| **IMP** | 0/59 (0.0) | 0/7 (0.0) | 0/3 (0.0) | 0/5 (0.0) | 0/1 (0.0) | 0/1 (0.0) | 0/3 (0.0) | 0/1 (0.0) | 0/1 (0.0) | | 0/11 (0.0) | 0/4 (0.0) | 0/2(0.0) |
| **TZP** | 1/55 (1.8) | 0/7 (0.0) | 0/3 (0.0) | 0/5 (0.0) | 0/1 (0.0) | 0/1 (0.0) | 0/3 (0.0) | 0/1 (0.0) | 0/1 (0.0) | | 0/11 (0.0) | 0/4 (0.0) | 0/2(0.0) |
| **CAZ** | 0/58 (0.0) | 0/7 (0.0) | 0/3 (0.0) | 0/5 (0.0) | 1/1 (100) | 0/1 (0.0) | 0/3 (0.0) | 0/1 (0.0) | 0/1 (0.0) | | 0/11 (0.0) | 0/4 (0.0) | 0/2(0.0) |
| **FEP** | 3/59 (5.1) | 0/7 (0.0) | 0/3 (0.0) | 0/5 (0.0) | 0/1 (0.0) | 0/1 (0.0) | 0/3 (0.0) | 0/1 (0.0) | 0/1 (0.0) | | 0/11 (0.0) | 0/4 (0.0) | 0/2(0.0) |
| **CIP** | 1/51 (2.0) | 0/7 (0.0) | 0/1 (0.0) | 0/5 (0.0) | 1/1 (100) | 0/1 (0.0) | 0/3 (0.0) | 0/1 (0.0) | 0/1 (0.0) | | 0/11 (0.0) | 0/4 (0.0) | 0/2(0.0) |
| **LVX** | 1/59 (1.7) | 0/7 (0.0) | 0/3 (0.0) | 0/5 (0.0) | 1/1 (100) | 0/1 (0.0) | 0/3 (0.0) | 0/1 (0.0) | 0/1 (0.0) | | 0/11 (0.0) | 0/4 (0.0) | 0/2(0.0) |
| **CST** | 47/53(88.7) | 7/7 (100) | 3/3 (100) | 0/5 (0.0) | 0/1 (0.0) | 1/1 (100) | 3/3 (100) | 1/1 (100) | 1/1 (100) | | 11/11 (100) | 4/4 (100) | 2/2 (100) |
| **TET** | 1/9 (11.1) | 0/0 (0.0) | 0/0 (0.0) | 0/5 (0.0) | 0/0 (0.0) | 0/0 (0.0) | 0/0 (0.0) | 0/0 (0.0) | 0/0 (0.0) | | 0/0 (0.0) | 0/0 (0.0) | 0/0 (0.0) |
