# Supplementary material for: Using comparative genomics to understand molecular features of carbapenem-resistant Acinetobacter baumannii from South Korea causing invasive infections and their clinical implications
Source: PLoS One. 2020 Feb 21;15(2):e0229416. doi: 10.1371/journal.pone.0229416 (PMC7034955; doi:10.1371/journal.pone.0229416)
Supplement: S5 Table — (DOCX) [file pone.0229416.s005.docx]

| **Supplementary Table S5.** Pairwise SNP distances within and between clades. | | | | | | | | |
| --- | --- | --- | --- | --- | --- | --- | --- | --- |
|  |  |  |  |  |  |  |  |  |
| **Clade** | **No. of strain** | **No. of pairs** | **Pairwise SNP distance statistics** | | | | | |
|  |  |  | **Min** | **1st Qu** | **Median** | **Mean** | **3rd Qu** | **Max** |
| 1 | 63 | 1953 | 39 | 1189 | 1291 | 1338 | 1459 | 3051 |
| 2 | 11 | 55 | 3515 | 6805 | 7565 | 7064 | 7600 | 7976 |
| 3 | 8 | 28 | 5519 | 5580 | 5995 | 5932 | 6250 | 6303 |
| 4 | 11 | 55 | 9015 | 9048 | 9066 | 9065 | 9080 | 9105 |
| Interclade | NA | 2662 | 2260 | 5767 | 8926 | 17414 | 10063 | 83304 |
| Sum | 98 | 4753 | 39 | 1350 | 5368 | 10524 | 9602 | 83304 |
