# Supplementary material for: Using comparative genomics to understand molecular features of carbapenem-resistant Acinetobacter baumannii from South Korea causing invasive infections and their clinical implications
Source: PLoS One. 2020 Feb 21;15(2):e0229416. doi: 10.1371/journal.pone.0229416 (PMC7034955; doi:10.1371/journal.pone.0229416)
Supplement: S6 Table — (DOCX) [file pone.0229416.s006.docx]

**Supplementary Table S6. Composition of virulence genes according to sequence types**

| Gene | Sequence types | | | | | | | | | | | | |
| --- | --- | --- | --- | --- | --- | --- | --- | --- | --- | --- | --- | --- | --- |
|  | ST191 (n=59) | ST357  (n=7) | ST368 (n=3) | ST208/ST1806 (n=5) | ST552 (n=1) | ST858 (n=1) | ST369  (n=3) | ST784 (n=1) | ST191/ST784 (n=1) | ST451/ST1809 (n=11) | ST447 (n=4) | | NA (n=2) |
| **Outer membrane vesicle** |  |  |  |  |  |  |  |  |  |  |  | |  |
| A1S_0009 | 59(100) | 7(100) | 3(100) | 5(100) | 1(100) | 1(100) | 3(100) | 1(100) | 1(100) | 11(100) | 4(100) | | 2(100) |
| A1S_0116 | **59(100)** | 7(100) | **0(0.0)** | 5(100) | 1(100) | 0(0.0) | 0(0.0) | 1(100) | 1(100) | 11(100) | **0(0.0)** | | 2(100) |
| A1S_1180 | 59(100) | 7(100) | 3(100) | 5(100) | 1(100) | 1(100) | 3(100) | 1(100) | 1(100) | 11(100) | 4(100) | | 2(100) |
| A1S_1321 | 58(98.3) | 7(100) | 3(100) | 5(100) | 1(100) | 1(100) | 3(100) | 1(100) | 1(100) | 11(100) | 4(100) | | 2(100) |
| A1S_1386 | 59(100) | 7(100) | 3(100) | 5(100) | 1(100) | 1(100) | 3(100) | 1(100) | 1(100) | 11(100) | 4(100) | | 2(100) |
| A1S_1510 | 59(100) | 7(100) | 3(100) | 5(100) | 1(100) | 1(100) | 3(100) | 1(100) | 1(100) | 11(100) | 4(100) | | 2(100) |
| A1S_1921 | 59(100) | 7(100) | 3(100) | 5(100) | 1(100) | 1(100) | 3(100) | 1(100) | 1(100) | 11(100) | 4(100) | | 2(100) |
| A1S_2470 | 59(100) | 7(100) | 3(100) | 5(100) | 1(100) | 1(100) | 3(100) | 1(100) | 1(100) | 11(100) | 4(100) | | 2(100) |
| A1S_2525 | 59(100) | 7(100) | 3(100) | 5(100) | 1(100) | 1(100) | 3(100) | 1(100) | 1(100) | 11(100) | 4(100) | | 2(100) |
| A1S_3143 | 59(100) | 7(100) | 3(100) | 5(100) | 1(100) | 1(100) | 3(100) | 1(100) | 1(100) | 11(100) | 4(100) | | 2(100) |
| A1S_3175 | 59(100) | 7(100) | 3(100) | 5(100) | 1(100) | 1(100) | 3(100) | 1(100) | 1(100) | 11(100) | 4(100) | | 2(100) |
| A1S_3411 | 59(100) | 7(100) | 3(100) | 5(100) | 1(100) | 1(100) | 3(100) | 1(100) | 1(100) | 11(100) | 4(100) | | 2(100) |
| *ACV72173.1* | 56(94.9) | 6(85.7) | **1(33.3)** | 4(80) | 1(100) | 1(100) | 3(100) | 1(100) | 1(100) | 11(100) | 4(100) | | 1(50) |
| *ACV72174.1* | 56(94.9) | 7(100) | **1(33.3)** | 4(80) | 1(100) | 1(100) | 3(100) | 1(100) | 1(100) | 11(100) | 4(100) | | 1(50) |
| *ADB23465.1* | 55(93.2) | 7(100) | **1(33.3)** | 4(80) | 1(100) | 1(100) | 3(100) | 1(100) | 1(100) | 11(100) | 4(100) | | 1(50) |
| *ADB23466.1* | 51(86.4) | 5(71.4) | 1(33.3) | **2(40.0)** | 1(100) | 1(100) | 3(100) | 1(100) | 1(100) | 10(90.9) | 4(100) | | 1(50) |
| *ADB23467.1* | 55(93.2) | 7(100) | **1(33.3)** | 4(80) | 1(100) | 1(100) | 3(100) | 1(100) | 0(0.0) | 11(100) | 4(100) | | 1(50) |
| *ADB23468.1* | 56(94.9) | 7(100) | **1(33.3)** | 4(80) | 1(100) | 1(100) | 3(100) | 1(100) | 1(100) | 11(100) | 4(100) | | 1(50) |
| *ADB23470.1* | 56(94.9) | 7(100) | **1(33.3)** | 4(80) | 1(100) | 1(100) | 3(100) | 1(100) | 1(100) | 11(100) | 4(100) | | 1(50) |
| *ADB23471.1* | 53(89.8) | 6(85.7) | **1(33.3)** | 4(80) | 1(100) | 1(100) | 3(100) | 1(100) | 1(100) | 11(100) | 4(100) | | 1(50) |
| *ADB23472.1* | 49(83.1) | 7(100) | 1(33.3) | 4(80) | 1(100) | 1(100) | 2(66.7) | 1(100) | 1(100) | 11(100) | 4(100) | | 1(50) |
| *ADB23473.1* | 55(93.2) | 7(100) | **1(33.3)** | 4(80) | 1(100) | 1(100) | 3(100) | 1(100) | 1(100) | 9(81.8) | 4(100) | | 1(50) |
| *GADB23474.1* | 56(94.9) | 6(85.7) | **1(33.3)** | 4(80) | 1(100) | 1(100) | 3(100) | 1(100) | 1(100) | 11(100) | 4(100) | | 1(50) |
| *GADB23475.1* | 56(94.9) | 7(100) | **1(33.3)** | 4(80) | 1(100) | 1(100) | 3(100) | 1(100) | 1(100) | 11(100) | 4(100) | | 1(50) |
| *bla*_OXA-24_ | 0(0.0) | 0(0.0) | 0(0.0) | 0(0.0) | 0(0.0) | 0(0.0) | 0(0.0) | 0(0.0) | 0(0.0) | 0(0.0) | 0(0.0) | | 0(0.0) |
| **Antibiotic resistance** |  |  |  |  |  |  |  |  |  |  |  | |  |
| ABUW_1156 | **59(100)** | 7(100) | 3(100) | 5(100) | 1(100) | 0(0.0) | 3(100) | 1(100) | 1(100) | 11(100) | **0(0.0)** | | 2(100) |
| ABUW_1499 | **56(94.9)** | **0(0.0)** | 3(100) | 5(100) | 1(100) | 1(100) | 1(33.3) | 1(100) | 1(100) | 11(100) | 2(50) | | 2(100) |
| ABUW_1520 | **54(91.5)** | **0(0.0)** | 3(100) | 5(100) | 1(100) | 1(100) | 1(33.3) | 1(100) | 1(100) | **0(0.0)** | 2(100) | | 2(100) |
| ABUW_1645 | 59(100) | 7(100) | 3(100) | 5(100) | 1(100) | 1(100) | 3(100) | 1(100) | 1(100) | 11(100) | 4(100) | | 2(100) |
| ABUW_1672 | 59(100) | 7(100) | 3(100) | 5(100) | 1(100) | 1(100) | 3(100) | 1(100) | 1(100) | 11(100) | 4(100) | | 2(100) |
| ABUW_1673 | 59(100) | 7(100) | 3(100) | 5(100) | 1(100) | 1(100) | 3(100) | 1(100) | 1(100) | 11(100) | 4(100) | | 2(100) |
| ABUW_1692 | 58(98.3) | 7(100) | 3(100) | 5(100) | 1(100) | 1(100) | 3(100) | 1(100) | 1(100) | 11(100) | 4(100) | | 2(100) |
| ABUW_1755 | 59(100) | 7(100) | 3(100) | 5(100) | 1(100) | 1(100) | 3(100) | 1(100) | 1(100) | 11(100) | 4(100) | | 2(100) |
| ABUW_1768 | 59(100) | 7(100) | 3(100) | 5(100) | 1(100) | 1(100) | 3(100) | 1(100) | 1(100) | 11(100) | 4(100) | | 2(100) |
| ABUW_1849 | 59(100) | 7(100) | 3(100) | 5(100) | 1(100) | 1(100) | 3(100) | 1(100) | 1(100) | 11(100) | 4(100) | | 2(100) |
| ABUW_1851 | 59(100) | 7(100) | 3(100) | 5(100) | 1(100) | 1(100) | 3(100) | 1(100) | 1(100) | 11(100) | 4(100) | | 2(100) |
| ABUW_1966 | 0(0.0) | 0(0.0) | 0(0.0) | 0(0.0) | 0(0.0) | 0(0.0) | 0(0.0) | 0(0.0) | 0(0.0) | 0(0.0) | 0(0.0) | | 0(0.0) |
| ABUW_2074 | 59(100) | 7(100) | 3(100) | 5(100) | 1(100) | 1(100) | 3(100) | 1(100) | 1(00) | 11(100) | 4(100) | | 2(100) |
| ABUW_2550 | 58(98.3) | 7(100) | 3(100) | 5(100) | 1(100) | 1(100) | 3(100) | 1(100) | 1(00) | 11(100) | 4(100) | | 2(100) |
| **Aromatic hydrocarbon metabolism** |  |  |  |  |  |  |  |  |  |  |  | |  |
| ABUW_2090 | 59(100) | 7(100) | 3(100) | 5(100) | 1(100) | 1(100) | 3(100) | 1(100) | 1(100) | 11(100) | 4(100) | | 2(100) |
| ABUW_2123 | 59(100) | 7(100) | 3(100) | 5(100) | 1(100) | 1(100) | 3(100) | 1(100) | 1(100) | 11(100) | 4(100) | | 2(100) |
| ABUW_2236 | 59(100) | 7(100) | 3(100) | 5(100) | 1(100) | 1(100) | 3(100) | 1(100) | 1(100) | 11(100) | 4(100) | | 2(100) |
| ABUW_2349 | 59(100) | 7(100) | 3(100) | 5(100) | 1(100) | 1(100) | 3(100) | 1(100) | 1(100) | 11(100) | 4(100) | | 2(100) |
| ABUW_2370 | 59(100) | 7(100) | 3(100) | 5(100) | 1(100) | 1(100) | 3(100) | 1(100) | 1(100) | 11(100) | 4(100) | | 2(100) |
| ABUW_2374 | 59(100) | 7(100) | 3(100) | 5(100) | 1(100) | 1(100) | 3(100) | 1(100) | 1(100) | 11(100) | 4(100) | | 2(100) |
| *benP1* | 59(100) | 7(100) | 3(100) | 5(100) | 1(100) | 1(100) | 3(100) | 1(100) | 1(100) | 11(100) | 4(100) | | 2(100) |
| *paaI1* | 59(100) | 7(100) | 3(100) | 5(100) | 1(100) | 1(100) | 3(100) | 1(100) | 1(100) | 11(100) | 4(100) | | 2(100) |
| *paaY* | 59(100) | 7(100) | 3(100) | 5(100) | 1(100) | 1(100) | 3(100) | 1(100) | 1(100) | 11(100) | 4(100) | | 2(100) |
| *pcaC* | 59(100) | 7(100) | 3(100) | 5(100) | 1(100) | 1(100) | 3(100) | 1(100) | 1(100) | 11(100) | 4(100) | | 2(100) |
| *pcaD1* | 59(100) | 7(100) | 3(100) | 5(100) | 1(100) | 1(100) | 3(100) | 1(100) | 1(100) | 11(100) | 4(100) | | 2(100) |
| *pcaU* | 59(100) | 7(100) | 3(100) | 5(100) | 1(100) | 1(100) | 3(100) | 1(100) | 1(100) | 11(100) | 4(100) | | 2(100) |
| **Transcriptional regulation** |  |  |  |  |  |  |  |  |  |  |  | |  |
| ABUW_2520 | 59(100) | 7(100) | 3(100) | 5(100) | 1(100) | 1(00) | 3(100) | 1(00) | 1(00) | 11(100) | 4(100) | | 2(100) |
| ABUW_2544 | 58(98.3) | 7(100) | 3(100) | 5(100) | 1(100) | 1(00) | 3(100) | 1(00) | 1(00) | 11(100) | 4(100) | | 2(100) |
| **Immune evasion** |  |  |  |  |  |  |  |  |  |  |  | |  |
| ACICU_00074 | 59(100) | 7(100) | 3(100) | 5(100) | 0(0.0) | 1(100) | 3(100) | 1(100) | 1(100) | 11(100) | 4(100) | | 2(100) |
| ACICU_00075 | **0(0.0)** | 0(0.0) | 0(0.0) | **5(100)** | 0(0.0) | 1(100) | 0(0.0) | 0(0.0) | 0(0.0) | **11(100)** | **0(0.0)** | | 0(0.0) |
| ACICU_00076 | **0(0.0)** | 0(0.0) | 0(0.0) | **5(100)** | 0(0.0) | 0(0.0) | 0(0.0) | 0(0.0) | 0(0.0) | **11(100)** | **0(0.0)** | | 0(0.0) |
| ACICU_00077 | **0(0.0)** | 0(0.0) | 0(0.0) | **5(100)** | 0(0.0) | 0(0.0) | 0(0.0) | 0(0.0) | 0(0.0) | **11(100)** | **0(0.0)** | | 0(0.0) |
| ACICU_00078 | **0(0.0)** | 0(0.0) | 0(0.0) | **5(100)** | 0(0.0) | 0(0.0) | 0(0.0) | 0(0.0) | 0(0.0) | **11(100)** | **0(0.0)** | | 0(0.0) |
| ACICU_00079 | **0(0.0)** | 0(0.0) | 0(0.0) | **5(100)** | 0(0.0) | 0(0.0) | 0(0.0) | 0(0.0) | 0(0.0) | **11(100)** | **0(0.0)** | | 0(0.0) |
| ACICU_00080 | **0(0.0)** | 0(0.0) | 0(0.0) | **5(100)** | 0(0.0) | 0(0.0) | 0(0.0) | 0(0.0) | 0(0.0) | **11(100)** | **0(0.0)** | | 0(0.0) |
| ACICU_00081 | **0(0.0)** | 0(0.0) | 0(0.0) | **5(100)** | 0(0.0) | 0(0.0) | 0(0.0) | 0(0.0) | 0(0.0) | 0(0.0) | **0(0.0)** | | 0(0.0) |
| ACICU_00082 | **0(0.0)** | 0(0.0) | 0(0.0) | **5(100)** | 0(0.0) | 0(0.0) | 0(0.0) | 0(0.0) | 0(0.0) | 0(0.0) | **0(0.0)** | | 0(0.0) |
| ACICU_00083 | **0(0.0)** | 0(0.0) | 0(0.0) | **5(100)** | 0(0.0) | 0(0.0) | 0(0.0) | 0(0.0) | 0(0.0) | 0(0.0) | **0(0.0)** | | 0(0.0) |
| ACICU_00084 | **0(0.0)** | 0(0.0) | 0(0.0) | **5(100)** | 0(0.0) | 0(0.0) | 0(0.0) | 0(0.0) | 0(0.0) | 0(0.0) | **0(0.0)** | | 0(0.0) |
| ACICU_00085 | **0(0.0)** | 0(0.0) | 0(0.0) | **5(100)** | 0(0.0) | 0(0.0) | 0(0.0) | 0(0.0) | 0(0.0) | 0(0.0) | **0(0.0)** | | 0(0.0) |
| ACICU_00086 | **0(0.0)** | 0(0.0) | **3(100)** | **5(100)** | 0(0.0) | 0(0.0) | 0(0.0) | 0(0.0) | 0(0.0) | **11(100)** | **0(0.0)** | | 0(0.0) |
| ACICU_00087 | **0(0.0)** | 0(0.0) | **3(100)** | **5(100)** | 0(0.0) | 0(0.0) | 0(0.0) | 0(0.0) | 0(0.0) | **11(100)** | **0(0.0)** | | 0(0.0) |
| ACICU_00088 | 59(100) | 7(100) | 3(100) | 5(100) | 1(100) | 1(100) | 3(100) | 1(100) | 1(100) | 11(100) | 4(100) | 2(100) | |
| ACICU_00089 | 59(100) | 7(100) | 3(100) | 5(100) | 1(100) | 1(100) | 3(100) | 1(100) | 1(100) | 11(100) | 4(100) | 2(100) | |
| ACICU_00091 | 59(100) | 7(100) | 3(100) | 5(100) | 1(100) | 1(100) | 3(100) | 1(100) | 1(100) | 11(100) | 4(100) | 2(100) | |
| ACICU_00092 | 59(100) | 7(100) | 3(100) | 5(100) | 1(100) | 1(100) | 3(100) | 1(100) | 1(100) | 11(100) | 4(100) | 2(100) | |
| *lpsB* | 59(100) | 7(100) | 3(100) | 5(100) | 1(100) | 1(100) | 3(100) | 1(100) | 1(100) | 11(100) | 4(100) | | 2(100) |
| *lptE* | 59(100) | 7(100) | 3(100) | 5(100) | 1(100) | 1(100) | 3(100) | 1(100) | 1(100) | 11(100) | 4(100) | | 2(100) |
| *lpxA* | 59(100) | 7(100) | 3(100) | 5(100) | 1(100) | 1(100) | 3(100) | 1(100) | 1(100) | 11(100) | 4(100) | | 2(100) |
| *lpxB* | 59(100) | 7(100) | 3(100) | 5(100) | 1(100) | 1(100) | 3(100) | 1(100) | 1(100) | 11(100) | 4(100) | | 2(100) |
| *lpxC* | 59(100) | 7(100) | 3(100) | 5(100) | 1(100) | 1(100) | 3(100) | 1(100) | 1(100) | 11(100) | 4(100) | | 2(100) |
| *lpxD* | 59(100) | 7(100) | 3(100) | 5(100) | 1(100) | 1(100) | 3(100) | 1(100) | 1(100) | 11(100) | 4(100) | | 2(100) |
| *lpxL* | 59(100) | 7(100) | 3(100) | 5(100) | 1(100) | 1(100) | 3(100) | 1(100) | 1(100) | 11(100) | 4(100) | | 2(100) |
| *lpxM* | 59(100) | 7(100) | 3(100) | 5(100) | 1(100) | 1(100) | 3(100) | 1(100) | 1(100) | 11(100) | 4(100) | | 2(100) |
| *eps* | **59(100)** | **0(0.0)** | **0(0.0)** | 5(100) | 1(100) | 1(100) | 3(100) | 1(100) | 1(100) | 11(100) | 4(100) | |  |
| *pgi* | 59(100) | 7(100) | 3(100) | 5(100) | 1(100) | 1(100) | 3(100) | 1(100) | 1(100) | 11(100) | 4(100) | | 2(100) |
| *ptk* | 59(100) | 7(100) | 3(100) | 5(100) | 1(100) | 1(100) | 3(100) | 1(100) | 1(100) | 11(100) | 4(100) | | 2(100) |
| *ptp* | 59(100) | 7(100) | 3(100) | 5(100) | 1(100) | 1(100) | 3(100) | 1(100) | 1(100) | 11(100) | 4(100) | | 2(100) |
| **Regulation** |  |  |  |  |  |  |  |  |  |  |  | |  |
| *abaI* | **59(100)** | 7(100) | **0(0.0)** | 5(100) | 1(100) | 0(0.0) | 3(100) | 1(100) | 1(100) | 11(100) | **0(0.0)** | | 2(100) |
| *abaR* | **54(91.5)** | 7(100) | **0(0.0)** | 5(100) | 1(100) | 0(0.0) | 3(100) | 1(100) | 1(100) | 11(100) | **0(0.0)** | | 2(100) |
| *bfmR* | 59(100) | 7(100) | 3(100) | 5(100) | 1(100) | 1(100) | 3(100) | 1(100) | 1(100) | 11(100) | 4(100) | | 2(100) |
| *bfmS* | 59(100) | 7(100) | 3(100) | 5(100) | 1(100) | 1(100) | 3(100) | 1(100) | 1(100) | 11(100) | 4(100) | | 2(100) |
| **Killing of host cells** |  |  |  |  |  |  |  |  |  |  |  | |  |
| *abeD* | 59(100) | 7(100) | 3(100) | 5(100) | 1(100) | 0(0.0) | 3(100) | 1(100) | 1(100) | 11(100) | 4(100) | | 2(100) |
| *envZ* | 59(100) | 7(100) | 3(100) | 5(100) | 1(100) | 1(100) | 3(100) | 1(100) | 1(100) | 11(100) | 4(100) | | 2(100) |
| *fhaB* | **58(98.3)** | 7(100) | 3(100) | 5(100) | 1(100) | 1(100) | 3(100) | 1(100) | 1(100) | 9(81.8) | **0(0.0)** | | 1(50) |
| *fhaC* | 0(0.0) | 0(0.0) | 0(0.0) | 0(0.0) | 0(0.0) | 0(0.0) | 0(0.0) | 0(0.0) | 0(0.0) | 0(0.0) | 0(0.0) | | 0(0.0) |
| **Biofilm formation** |  |  |  |  |  |  |  |  |  |  |  | |  |
| *adeF* | 59(100) | 7(100) | 3(100) | 5(100) | 1(100) | 1(100) | 3(100) | 1(100) | 1(100) | 11(100) | 4(100) | | 2(100) |
| *adeG* | 59(100) | 7(100) | 3(100) | 5(100) | 1(100) | 1(100) | 3(100) | 1(100) | 1(100) | 11(100) | 4(100) | | 2(100) |
| *bap* | **59(100)** | 7(100) | 3(100) | 5(100) | 1(100) | 0(0.0) | 3(100) | 1(100) | 1(100) | 11(100) | 4(100) | | 2(100) |
| *csuA* | **57(96.6)** | **0(0.0)** | 3(100) | 5(100) | 1(100) | 0(0.0) | 1(33.3) | 1(100) | 1(100) | 10(90.9) | **1(25)** | | 2(100) |
| *csuA/B* | **57(96.6)** | **0(0.0)** | 3(100) | 5(100) | 1(100) | 0(0.0) | 1(33.3) | 1(100) | 1(100) | 9(81.8) | **1(25)** | | 2(100) |
| *csuB* | **57(96.6)** | **0(0.0)** | 3(100) | 5(100) | 1(100) | 0(0.0) | 1(33.3) | 1(100) | 1(100) | 9(81.8) | **1(25)** | | 2(100) |
| *csuC* | **57(96.6)** | **0(0.0)** | 3(100) | 5(100) | 1(100) | 0(0.0) | 1(33.3) | 1(100) | 1(100) | 10(90.9) | **1(25)** | | 2(100) |
| *csuD* | **57(96.6)** | **0(0.0)** | 3(100) | 5(100) | 1(100) | 0(0.0) | 1(33.3) | 1(100) | 1(100) | 10(90.9) | **1(25)** | | 2(100) |
| *csuE* | **56(94.9)** | **0(0.0)** | 3(100) | 5(100) | 1(100) | 0(0.0) | 1(33.3) | 1(100) | 1(100) | 10(90.9) | **1(25)** | | 2(100) |
| *pgaA* | 59(100) | 7(100) | 3(100) | 5(100) | 1(100) | 1(100) | 3(100) | 1(100) | 1(100) | 11(100) | 4(100) | | 2(100) |
| *pgaB* | 59(100) | 7(100) | 3(100) | 5(100) | 1(100) | 1(100) | 3(100) | 1(100) | 1(100) | 11(100) | 4(100) | | 2(100) |
| *pgaC* | **59(100)** | 7(100) | 3(100) | 5(100) | 1(100) | 0(0.0) | 3(100) | 1(100) | 1(100) | 11(100) | **0(0.0)** | | 2(100) |
| *pgaD* | **0(0.0)** | **7(100)** | 2(66.7) | **5(100)** | 0(0.0) | 1(100) | **3(100)** | 0(0.0) | 0(0.0) | **11(100)** | **4(100)** | | 0(0.0) |
| *adeH* | 59(100) | 7(100) | 3(100) | 5(100) | 1(100) | 1(100) | 3(100) | 1(100) | 1(100) | 11(100) | 4(100) | | 2(100) |
| **Antibiotic resistance** |  |  |  |  |  |  |  |  |  |  |  | |  |
| *adeI* | 59(100) | 7(100) | 3(100) | 5(100) | 1(100) | 1(100) | 3(100) | 1(100) | 1(100) | 11(100) | 4(100) | | 2(100) |
| *adeJ* | 59(100) | 7(100) | 3(100) | 5(100) | 1(100) | 1(100) | 3(100) | 1(100) | 1(100) | 11(100) | 4(100) | | 2(100) |
| *adeK* | 59(100) | 7(100) | 3(100) | 5(100) | 1(100) | 1(100) | 3(100) | 1(100) | 1(100) | 11(100) | 4(100) | | 2(100) |
| *bla*_PER-1_ | **0(0.0)** | 0(0.0) | 0(0.0) | **4(80.0)** | 0(0.0) | 0(0.0) | 0(0.0) | 0(0.0) | 0(0.0) | 0(0.0) | 0(0.0) | | 0(0.0) |
| **Transcriptional regulation** |  |  |  |  |  |  |  |  |  |  |  | |  |
| *alkR* | 59(100) | 7(100) | 3(100) | 5(100) | 1(100) | 1(100) | 3(100) | 1(100) | 1(100) | 11(100) | 4(100) | | 2(100) |
| *gigA* | 59(100) | 7(100) | 3(100) | 5(100) | 1(100) | 1(100) | 3(100) | 1(100) | 1(100) | 11(100) | 4(100) | | 2(100) |
| *gigB* | 59(100) | 7(100) | 3(100) | 5(100) | 1(100) | 1(100) | 3(100) | 1(100) | 1(100) | 11(100) | 4(100) | | 2(100) |
| *gigC* | 59(100) | 7(100) | 3(100) | 5(100) | 1(100) | 1(100) | 3(100) | 1(100) | 1(100) | 11(100) | 4(100) | | 2(100) |
| *soxR* | 58(98.3) | 7(100) | 3(100) | 5(100) | 1(100) | 1(100) | 3(100) | 1(100) | 1(100) | 11(100) | 4(100) | | 2(100) |
| **Iron uptake** |  |  |  |  |  |  |  |  |  |  |  | |  |
| *barA* | 59(100) | 7(100) | 3(100) | 5(100) | 1(100) | 0(0.0) | 3(100) | 1(100) | 1(100) | 11(100) | 4(100) | | 2(100) |
| *barB* | 59(100) | 7(100) | 3(100) | 5(100) | 1(100) | 1(100) | 3(100) | 1(100) | 1(100) | 11(100) | 4(100) | | 2(100) |
| *basA* | 59(100) | 7(100) | 3(100) | 5(100) | 1(100) | 1(100) | 3(100) | 1(100) | 1(100) | 11(100) | 4(100) | | 2(100) |
| *basB* | 59(100) | 7(100) | 3(100) | 5(100) | 1(100) | 0(0.0) | 3(100) | 1(100) | 1(100) | 11(100) | 4(100) | | 2(100) |
| *basC* | 59(100) | 7(100) | 3(100) | 5(100) | 1(100) | 1(100) | 3(100) | 1(100) | 1(100) | 11(100) | 4(100) | | 2(100) |
| *basD* | 59(100) | 7(100) | 3(100) | 5(100) | 1(100) | 1(100) | 3(100) | 1(100) | 1(100) | 11(100) | 4(100) | | 2(100) |
| *basF* | 59(100) | 7(100) | 3(100) | 5(100) | 1(100) | 1(100) | 3(100) | 1(100) | 1(100) | 11(100) | 4(100) | | 2(100) |
| *basG* | 59(100) | 7(100) | 3(100) | 5(100) | 1(100) | 1(100) | 3(100) | 1(100) | 1(100) | 11(100) | 4(100) | | 2(100) |
| *basH* | 59(100) | 7(100) | 3(100) | 5(100) | 1(100) | 1(100) | 3(100) | 1(100) | 1(100) | 11(100) | 4(100) | | 2(100) |
| *basI* | 59(100) | 7(100) | 3(100) | 5(100) | 1(100) | 1(100) | 3(100) | 1(100) | 1(100) | 11(100) | 4(100) | | 2(100) |
| *basJ* | 59(100) | 7(100) | 3(100) | 5(100) | 1(100) | 1(100) | 3(100) | 1(100) | 1(100) | 11(100) | 4(100) | | 2(100) |
| *bauA* | **59(100)** | 7(100) | 3(100) | 5(100) | 1(100) | 1(100) | 3(100) | 1(100) | 1(100) | 11(100) | **0(0.0)** | | 2(100) |
| *bauB* | 59(100) | 7(100) | 3(100) | 5(100) | 1(100) | 1(100) | 3(100) | 1(100) | 1(100) | 11(100) | 4(100) | | 2(100) |
| *bauC* | 59(100) | 7(100) | 3(100) | 5(100) | 1(100) | 1(100) | 3(100) | 1(100) | 1(100) | 11(100) | 4(100) | | 2(100) |
| *bauD* | 59(100) | 7(100) | 3(100) | 5(100) | 1(100) | 1(100) | 3(100) | 1(100) | 1(100) | 11(100) | 4(100) | | 2(100) |
| *bauE* | 59(100) | 7(100) | 3(100) | 5(100) | 1(100) | 1(100) | 3(100) | 1(100) | 1(100) | 11(100) | 4(100) | | 2(100) |
| *bauF* | 59(100) | 7(100) | 3(100) | 5(100) | 1(100) | 1(100) | 3(100) | 1(100) | 1(100) | 11(100) | 4(100) | | 2(100) |
| *nfuA* | 59(100) | 7(100) | 3(100) | 5(100) | 1(100) | 1(100) | 3(100) | 1(100) | 1(100) | 11(100) | 4(100) | | 2(100) |
| **Porin** |  |  |  |  |  |  |  |  |  |  |  | |  |
| *carO* | 57(96.6) | 7(100) | 3(100) | 5(100) | 1(100) | 1(100) | **1(33.3)** | 1(100) | 1(100) | 11(100) | 4(100) | | 2(100) |
| *omp22* | 59(100) | 7(100) | 3(100) | 5(100) | 1(100) | 1(100) | 3(100) | 1(100) | 1(100) | 11(100) | 4(100) | | 2(100) |
| *omp33-36* | 0(0.0) | 0(0.0) | 0(0.0) | 0(0.0) | 0(0.0) | 0(0.0) | 0(0.0) | 0(0.0) | 0(0.0) | 0(0.0) | 0(0.0) | | 0(0.0) |
| *ompR* | 59(100) | 7(100) | 3(100) | 5(100) | 1(100) | 1(100) | 3(100) | 1(100) | 1(100) | 11(100) | 4(100) | | 2(100) |
| *orpD* | 59(100) | 7(100) | 3(100) | 5(100) | 1(100) | 1(100) | 3(100) | 1(100) | 1(100) | 11(100) | 4(100) | | 2(100) |
| **Serun resistance, invasion** |  |  |  |  |  |  |  |  |  |  |  | |  |
| *cipA* | 59(100) | 7(100) | 3(100) | 5(100) | 1(100) | 1(100) | 3(100) | 1(100) | 1(100) | 11(100) | 4(100) | | 2(100) |
| *cobA* | 59(100) | 7(100) | 3(100) | 5(100) | 1(100) | 1(100) | 3(100) | 1(100) | 1(100) | 11(100) | 4(100) | | 2(100) |
| *pbpG* | 59(100) | 7(100) | 3(100) | 5(100) | 1(100) | 1(100) | 3(100) | 1(100) | 1(100) | 11(100) | 4(100) | | 2(100) |
| *surA1* | 59(100) | 7(100) | 3(100) | 5(100) | 1(100) | 1(100) | 3(100) | 1(100) | 1(100) | 11(100) | 4(100) | | 2(100) |
| *tuf* | 58(98.3) | 7(100) | 3(100) | 5(100) | 1(100) | 1(100) | 3(100) | 1(100) | 1(100) | 11(100) | 4(100) | | 2(100) |
| *typA* | 59(100) | 7(100) | 3(100) | 5(100) | 1(100) | 1(100) | 3(100) | 1(100) | 1(100) | 11(100) | 4(100) | | 2(100) |
| **Enzyme** |  |  |  |  |  |  |  |  |  |  |  | |  |
| *plcC1* | **0(0.0)** | **0(0.0)** | 0(0.0) | 0(0.0) | 0(0.0) | 1(00) | 0(0.0) | 0(0.0) | 0(0.0) | **11(100)** | 0(0.0) | | 0(0.0) |
| *plcC2* | 59(100) | 7(100) | 3(100) | 5(100) | 1(100) | 1(100) | 3(100) | 1(100) | 1(100) | 11(100) | 4(100) | | 2(100) |
| *plcD* | 59(100) | 7(100) | 3(100) | 5(100) | 1(100) | 1(100) | 3(100) | 1(100) | 1(100) | 11(100) | 4(100) | | 2(100) |
| *pldA* | 59(100) | 7(100) | 3(100) | 5(100) | 1(100) | 1(100) | 3(100) | 1(100) | 1(100) | 11(100) | 4(100) | | 2(100) |
| **Cysteine metabolism** |  |  |  |  |  |  |  |  |  |  |  | |  |
| *cysD* | 59(100) | 7(100) | 3(100) | 5(100) | 1(100) | 1(100) | 3(100) | 1(100) | 1(100) | 11(100) | 4(100) | | 2(100) |
| *cysE* | 59(100) | 7(100) | 3(100) | 5(100) | 1(100) | 1(100) | 3(100) | 1(100) | 1(100) | 11(100) | 4(100) | | 2(100) |
| *cysH* | 59(100) | 7(100) | 3(100) | 5(100) | 1(100) | 1(100) | 3(100) | 1(100) | 1(100) | 11(100) | 4(100) | | 2(100) |
| *cysI* | 59(100) | 7(100) | 3(100) | 5(100) | 1(100) | 1(100) | 3(100) | 1(100) | 1(100) | 11(100) | 4(100) | | 2(100) |
| *cysN* | 59(100) | 7(100) | 3(100) | 5(100) | 1(100) | 1(100) | 3(100) | 1(100) | 1(100) | 11(100) | 4(100) | | 2(100) |
| *cysQ* | 59(100) | 7(100) | 3(100) | 5(100) | 1(100) | 1(100) | 3(100) | 1(100) | 1(100) | 11(100) | 4(100) | | 2(100) |
| *sulP* | 59(100) | 7(100) | 3(100) | 5(100) | 1(100) | 1(100) | 3(100) | 1(100) | 1(100) | 11(100) | 4(100) | | 2(100) |
| **Siderophore biosynthesis** |  |  |  |  |  |  |  |  |  |  |  | |  |
| *entA* | 59(100) | 7(100) | 3(100) | 5(100) | 1(100) | 1(100) | 3(100) | 1(100) | 1(100) | 11(100) | 4(100) | | 2(100) |
|  |  |  |  |  |  |  |  |  |  |  |  | |  |
| **Neutrophil influx** |  |  |  |  |  |  |  |  |  |  |  | |  |
| *gacS* | 59(100) | 7(100) | 3(100) | 5(100) | 1(100) | 0(0.0) | 3(100) | 1(00) | 1(00) | **9(81.8)** | 4(100) | | 2(100) |
| *paaA* | 58(98.3) | 7(100) | 3(100) | 5(100) | 1(100) | 1(100) | 3(100) | 1(100) | 1(100) | 11(100) | 4(100) | | 2(100) |
| **Type II protein secretion system** |  |  |  |  |  |  |  |  |  |  |  | |  |
| *gspD* | 59(100) | 7(100) | 3(100) | 5(100) | 1(100) | 1(100) | 3(100) | 1(100) | 1(100) | 11(100) | 4(100) | | 2(100) |
|  |  |  |  |  |  |  |  |  |  |  |  | |  |
| **Type VI protein secretion system** |  |  |  |  |  |  |  |  |  |  |  | |  |
| *vgrG1* | **59(100)** | **0(0.0)** | **0(0.0)** | **0(0.0)** | 1(100) | 0(0.0) | 3(100) | 1(100) | 1(100) | **0(0.0)** | **0(0.0)** | | 2(100) |
| *vgrG2* | **59(100)** | 7(100) | **1(33.3)** | 5(100) | 1(100) | 1(00) | 3(100) | 1(100) | 1(100) | **0(0.0)** | 4(100) | | 2(100) |
| *vgrG3* | 59(100) | 7(100) | 3(100) | 5(100) | 1(100) | 1(00) | 3(100) | 1(100) | 1(100) | 11(100) | 4(100) | | 2(100) |
| *vgrG4* | **59(100)** | **0(0.0)** | **0(0.0)** | **0(0.0)** | 1(100) | 0(0.0) | 3(100) | 1(100) | 1(100) | **0(0.0)** | **0(0.0)** | | 2(100) |
| *hcp* | **0(0.0)** | **7(100)** | 0(0.0) | **5(100)** | 0(0.0) | 0(0.0) | **3(100)** | 0(0.0) | 1(100) | **11(100)** | 0(0.0) | | 0(0.0) |
| **Type V protein secretion system** |  |  |  |  |  |  |  |  |  |  |  | |  |
| *ata* | **53(89.8)** | **2(28.6)** | 3(100) | 5(100) | 1(100) | 1(00) | 3(100) | 1(00) | 1(00) | 11(100) | 4(100) | | 1(50) |
| **Stress response genes** |  |  |  |  |  |  |  |  |  |  |  | |  |
| *kef* | 59(100) | 7(100) | 3(100) | 5(100) | 1(100) | 1(00) | 3(100) | 1(00) | 0(0.0) | 11(100) | 4(100) | | 2(100) |
| *kefF* | 58(98.3) | 7(100) | **1(33.3)** | 4(80) | 1(100) | 1(00) | 3(100) | 1(100) | 1(100) | 11(100) | 4(100) | | 2(100) |
| *mscS* | 59(100) | 7(100) | 3(100) | 5(100) | 1(100) | 1(00) | 3(100) | 1(100) | 1(100) | 11(100) | 4(100) | | 2(100) |
| *ostB* | 59(100) | 7(100) | 3(100) | 5(100) | 1(100) | 1(00) | 3(100) | 1(100) | 1(100) | 11(100) | 4(100) | | 2(100) |
| *recA* | 59(100) | 7(100) | 3(100) | 5(100) | 1(100) | 1(00) | 3(100) | 1(100) | 1(100) | 11(100) | 4(100) | | 2(100) |
| *resP* | 59(100) | 7(100) | 3(100) | 5(100) | 1(100) | 1(00) | 3(100) | 1(100) | 1(100) | 11(100) | 4(100) | | 2(100) |
| *trkH* | 59(100) | 7(100) | 3(100) | 5(100) | 1(100) | 1(00) | 3(100) | 1(100) | 1(100) | 11(100) | 4(100) | | 2(100) |
| *upsA* | 59(100) | 7(100) | 3(100) | 5(100) | 1(100) | 1(00) | 3(100) | 1(100) | 1(100) | 11(100) | 4(100) | | 2(100) |
| *uspA* | 0(0.0) | 0(0.0) | 0(0.0) | 0(0.0) | 0(0.0) | 0(0.0) | 0(0.0) | 0(0.0) | 0(0.0) | 0(0.0) | 0(0.0) | | 0(0.0) |
| *uvrD* | 0(0.0) | 0(0.0) | 0(0.0) | 0(0.0) | 0(0.0) | 0(0.0) | 0(0.0) | 0(0.0) | 0(0.0) | 0(0.0) | 0(0.0) | | 0(0.0) |
| **Manganese acquisition system** |  |  |  |  |  |  |  |  |  |  |  | |  |
| *mumC* | 58(98.3) | 7(100) | 3(100) | 4(80) | 1(100) | 1(00) | 3(100) | 1(00) | 1(00) | 11(100) | 4(100) | | 2(100) |
| *mumT* | 58(98.3) | 7(100) | 3(100) | 4(80) | 1(100) | 1(00) | 3(100) | 1(00) | 1(00) | 11(100) | 4(100) | | 2(100) |
| **Adherence** |  |  |  |  |  |  |  |  |  |  |  | |  |
| *ompA* | 59(100) | 7(100) | 3(100) | 5(100) | 1(010) | 1(00) | 3(100) | 1(00) | 1(00) | 11(100) | 4(100) | | 2(100) |
| **Micronutrient acquisition** |  |  |  |  |  |  |  |  |  |  |  | |  |
| *znuA* | 59(100) | 7(100) | 3(100) | 5(100) | 1(100) | 1(100) | 1(100) | 1(100) | 1(100) | 11(100) | 4(100) | | 2(100) |
| *znuB* | 59(100) | 7(100) | 3(100) | 5(100) | 1(100) | 1(100) | 1(100) | 1(100) | 1(100) | 11(100) | 4(100) | | 2(100) |
| *znuC* | 59(100) | 7(100) | 3(100) | 5(100) | 1(100) | 1(100) | 1(100) | 1(100) | 1(100) | 11(100) | 4(100) | | 2(100) |
| *zur* | 59(100) | 7(100) | 3(100) | 5(100) | 1(100) | 1(100) | 1(100) | 1(100) | 1(100) | 11(100) | 4(100) | | 2(100) |

Data are expressed as N (%).Values with statistical significance (p-value<0.05) when compared to groups of the rest were expressed in boldface. Abbreviation: ST, sequence type; NA, not applicable
