# Supplementary material for: Using comparative genomics to understand molecular features of carbapenem-resistant Acinetobacter baumannii from South Korea causing invasive infections and their clinical implications
Source: PLoS One. 2020 Feb 21;15(2):e0229416. doi: 10.1371/journal.pone.0229416 (PMC7034955; doi:10.1371/journal.pone.0229416)
Supplement: S7 Table — (DOCX) [file pone.0229416.s007.docx]

**Supplementary Table S7. Differences in distribution of insertion sequences according to sequence types**

| **Insertion Sequence** | Sequence type | | |  | Total |
| --- | --- | --- | --- | --- | --- |
|  | ST447(n=4) | ST451/ST1809(n=11) | ST191(n=59) | Other(n=24) |  |
| **IS*1** | **0(0.0)*** | 11(100.0) | 53(91.4) | 21(87.5) | 85(86.7) |
| **IS*2** | **0(0.0)*** | 11(100.0) | **59(100)*** | 23(95.8) | 93(94.9) |
| **IS*3** | **0(0.0)*** | 11(100.0) | 52(88.1) | 22(91.7) | 85(86.7) |
| **IS*4** | **0(0.0)*** | 11(100.0) | 51(86.4) | 21(87.5) | 83(84.7) |
| **IS*5** | **0(0.0)*** | **2(18.2)*** | **54(91.5)*** | 13(54.2) | 69(70.4) |
| **IS*6** | **0(0.0)*** | 11(100.0) | 56(96.6) | 23(95.8) | 90(91.8) |
| **IS*7** | 3(75) | **5(45.5)*** | **52(88.1)*** | 17(70.8) | 77(78.6) |

Data of sequence types only with statistical significance are shown. Data are expressed as N (%).

*signifies having statistical significance with p-value<0.05 when compared to rest of the groups
